# IL-21R-STAT3 signalling initiates a differentiation program in uterine tissue-resident NK cells to support pregnancy

**DOI:** 10.1038/s41467-023-42990-0

**Published:** 2023-11-04

**Authors:** Mengwei Han, Luni Hu, Di Wu, Yime Zhang, Peng Li, Xingyu Zhao, Yanyu Zeng, Guanqun Ren, Zhiyuan Hou, Yanli Pang, Tongbiao Zhao, Chao Zhong

**Affiliations:** 1 Institute of Systems Biomedicine, School of Basic Medical Sciences, Beijing Key Laboratory of Tumor Systems Biology, Peking University Health Science Center, 38 Xueyuan Road, Haidian District, Beijing, 100191 China; 2 Center for Reproductive Medicine, Department of Obstetrics and Gynecology, Peking University Third Hospital, Beijing, China; 3 National Stem Cell Resource Center, State Key Laboratory of Stem Cell and Reproductive Biology, Institute of Zoology, Institute for Stem Cell and Regeneration, Chinese Academy of Sciences, Beijing, China; 4 NHC Key Laboratory of Medical Immunology, Peking University, Beijing, 100191 China; 5 Key Laboratory of Molecular Immunology, Chinese Academy of Medical Sciences, Beijing, 100191 China

**Keywords:** NK cells, Cell death and immune response, Intrauterine growth, RNA sequencing

## Abstract

Tissue-resident Natural Killer (trNK) cells are crucial components of local immunity that activate rapidly upon infection. However, under steady state conditions, their responses are tightly controlled to prevent unwanted tissue damage. The mechanisms governing their differentiation and activation are not fully understood. Here, we characterise uterine trNK cells longitudinally during pregnancy by single cell RNA sequencing and find that the combined expression pattern of 4-1BB and CD55 defines their three distinct stages of differentiation in mice. Mechanistically, an IL-21R-STAT3 axis is essential for initiating the trNK cell differentiation. The fully differentiated trNK cells demonstrate enhanced functionality, which is necessary for remodelling spiral arteries in the decidua. We identify an apoptotic program that is specific to the terminal differentiation stage, which may preclude tissue damage by these highly activated trNK cells. In summary, uterine trNK cells become intensely active and effective during pregnancy, but tightly controlled via a differentiation program that also limits potential harm, suggesting an intricate mechanism for harnessing trNK cells in maintaining pregnancy.

## Abstract (web summary)

Uterine natural killer (NK) cells support tissue homeostasis in the uterus during pregnancy, but it is not fully known how they differentiate into potentially cytotoxic effector cells while avoiding tissue damage. Here authors show that Il21 receptor signalling via STAT3 activation governs their differentiation, while an apoptotic cell death program ensures that harm is limited.

## Introduction

Natural killer (NK) cells play critical roles in innate immunity primarily by secreting cytotoxic granules containing perforin and granzymes^1^. Recently, tissue-resident NK (trNK) cells, a distinct subset residing in peripheral tissues, have been discovered^2–6^. Rather than entering circulation, they remain within their tissues of residence. There, they are essential for maintaining tissue homeostasis^6,7^. In addition, type 1 innate lymphoid cells (ILC1s), a third population, also exhibit tissue residency but specialize in cytokine production compared to NK cells^8,9^. Conventional NK (cNK) cells, trNK cells, and ILC1s are all characterized by expression of NK1.1 and NKp46 on their cell surface. In addition, trNK cells also express certain markers, such as CD49a, but lack the mature cNK cell marker DX5^2,5,10^. In contrast, ILC1s, distinct from the two NK cell subsets, are distinguished by the absence of Eomes expression^8,11^.

Tissue-resident immune cells, including trNK cells, are the most critical components of local immunity^12^. Upon pathogen invasion, trNK cells rapidly activate to provide the first line of defense for their resident tissues^13^. Furthermore, trNK cells potently activate during specific physiological processes. For example, uterine trNK cells become profoundly activated during pregnancy, facilitating remodeling of spiral arteries in the decidua and supplying growth-promoting factors to the fetus^14,15^. TrNK cells demonstrate both tissue-resident and innate immune properties. They activate independently of antigen recognition, enabling an immediate response to inflammatory stimulation. However, to prevent unwanted overactivation and resultant tissue damage, trNK cell activation also requires tight restriction during physiological processes like pregnancy. The mechanisms regulating trNK cell activation under varying conditions remain elusive.

As abovementioned, a representative example of trNK cell activation under physiological conditions occurs in the uterus during pregnancy. During early decidualization, uterine trNK cells substantially activate and expand, becoming the predominant immune population at the maternal-fetal interface^3,16^. To ensure reproductive success, uterine trNK cell activation must be tightly regulated. Either dampening or enhancing uterine trNK cell activation elevates the risk of miscarriage^17^. Activated uterine trNK cells facilitate deep trophoblast invasion, enabling remodeling of spiral arteries by replacing the endothelial and smooth muscle linings of uterine arterioles^18–20^. Successful remodeling of uterine arteries guarantees adequate nutrient supply to the fetus^21^, whereas abnormal remodeling frequently results in fetal growth restriction or miscarriage^22–24^. For instance, deficiencies in uterine trNK cells of *Il15*^-/-^ and *Nfil3*^-/-^ mice, arising from developmental defects, have been shown to induce abnormal uterine artery remodeling and miscarriage^14,25,26^. Moreover, activated uterine trNK cells also produce growth-promoting factors such as pleiotrophin, osteoglycin, and osteopontin, which are indispensable for fetal growth^14^. Collectively, uterine trNK cell activation during pregnancy ensures reproductive success.

Intriguingly, uterine trNK cell and cNK cell activation differ markedly during pregnancy. While uterine trNK cells spring vigorously into action, cNK cells in the uterus remain quiescent^3,14^. Thus, uterine trNK cell activation likely relies upon distinct regulatory mechanisms during pregnancy. NK cell activation can be induced by various proinflammatory cytokines, including interleukin-2 (IL-2), IL-12, IL-15, IL-18, and IL-21. These cytokines activate Janus kinase (JAK)/signal transducer and activator of transcription (STAT) signaling pathways that potently activate NK cells^27–29^. The distinct tissue expression profiles of these proinflammatory cytokines suggest that they may preferentially fine-tune activation of uterine trNK cells^30,31^. Moreover, NK cell activation is also governed by NK activating and inhibitory receptors, as well as certain key transcriptional regulators^32–36^. Nonetheless, the mechanisms by which these factors regulate uterine trNK cell activation during pregnancy remain to be thoroughly elucidated.

In this study, we find that the IL-21 receptor (IL-21R) is selectively expressed by uterine trNK compared to cNK cells. Moreover, IL-21 signaling is activated during pregnancy, leading to a STAT3-dependent differentiation process that fine-tunes the effector function of uterine trNK. These findings provide insights into a previously unrecognized differentiation program of uterine trNK cells that plays a crucial role in supporting healthy pregnancy.

## Results

### Transcriptional profiling reveals a dynamic change of uterine trNK during decidualization

**Fig. 1:**
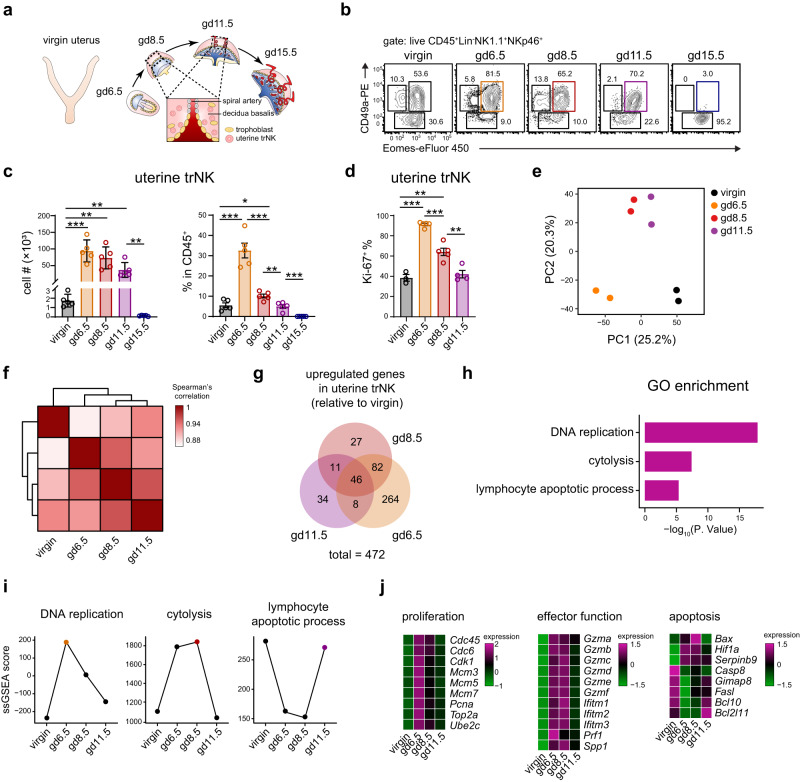
Profiling of transcriptomic changes in uterine trNK during decidualization. **a** Schematic diagram showing the change of uterus during pregnancy. The diagram in the bottom depicts the interaction between uterine trNK and trophoblasts which promotes spiral artery remodeling in decidua basalis. **b** Flow cytometry showing dynamic changes of uterine trNK (Eomes^+^CD49a^+^), cNK (Eomes^+^CD49a^-^), and ILC1 (Eomes^-^CD49a^+^) in virgin mice and during pregnancy (from gd6.5 to gd15.5). Overall NK/ILC1 cells are gated by live CD45^+^Lin (CD3/CD19/CD5/Gr-1)^-^NK1.1^+^NKp46^+^. **c** Cell number and percentage (in CD45^+^ immunocytes) of uterine trNK from virgin and pregnant (from gd6.5 to gd15.5) mice (*n* = 5 per group; ***P = 0.0002, **P = 0.0013, **P = 0.0074, **P = 0.0059; ***P = 0.0001, *P = 0.0155, ***P = 0.0003, **P = 0.0047, ***P = 0.0007). **d** Percentage of Ki67^+^ uterine trNK from virgin and pregnant (from gd6.5 to gd11.5) mice (*n* = 3, 5, 5, and 4 per group; ***P < 0.0001, **P = 0.0032, ***P = 0.0001, **P = 0.0041). **e** Principal component analysis (PCA) of uterine trNK from virgin mice and pregnant mice during decidualization (gd6.5, gd8.5, and gd11.5) (*n* = 2 per group). **f** Spearman’s correlation between uterine trNK from virgin mice and pregnant mice during decidualization (gd6.5, gd8.5, and gd11.5) (*n* = 2 per group). **g** Venn diagram showing total upregulated genes in uterine trNK during decidualization (related to Supplementary Fig. 1h). **h** Gene ontology (GO) analysis of the total upregulated genes in uterine trNK (**g**). **i** Single-sample GSEA (ssGSEA) scoring of uterine trNK at the indicated gestational days using the GO terms enriched in (**h**). **j** Heatmap graph showing relative expression changes of the representative genes in the enriched terms in uterine trNK at the indicated gestational days. Numbers indicate the percentages in each box. Data are shown as the mean ± SEM. *P*-values are calculated by two-sided unpaired *t*-test, *P < 0.05, **P < 0.01, ***P < 0.001. Data are representative of at least three independent experiments (**b**–**d**) or two independent experiments (**e**–**j**). Source data are provided as a Source Data file.

Among the three NK1.1^+^NKp46^+^ NK/ILC1 subpopulations in the uterus (Supplementary Fig. 1a, b), trNK represented the predominant subset that activated and expanded during pregnancy^14^. Consistent with previous reports, uterine trNK cells had substantially proliferated and exhibited elevated Ki-67 expression during decidualization (gestational days (gd) 6.5 to 11.5) (Fig. 1a–d)^3,37^. In contrast, though cNK cells and ILC1s exhibited a slight increase, they remained minor subpopulations among the CD45^+^ immune cells (Supplementary Fig. 1c, d). Proliferating uterine trNK cells played a critical role in trophoblast invasion and spiral artery remodeling during decidualization^21^. Afterwards, they rapidly declined and were nearly absent during the late gestational stage (gd15.5), presumably to avoid endangering the pregnancy (Fig. 1b, c). In line with a previous study^25^, proliferating uterine trNK cells also displayed enlarged cell sizes and increased cellular components, as indicated by their elevated forward and side scatter (FSC-A and SSC-A) parameters during flow cytometry examination (Supplementary Fig. 1e). These changes evidenced an immune cell activation^38^.

While the activation and function of trNK cells could be accurately demonstrated through their effector gene expression, there had been a lack of comprehensive study on the dynamic transcriptional profiling of uterine trNK cells during decidualization. Therefore, to further elucidate their activation during decidualization, we conducted an RNA-Sequencing (RNA-Seq) analysis using sort-purified uterine trNK at different gestational days (Supplementary Fig. 1f). Post-sorting examination confirmed that the purity of the uterine trNK cells exceeded 95% (Supplementary Fig. 1g). Through principal component analysis (PCA) and Spearman’s correlation, it was evident that the uterine trNK at gestational days 6.5, 8.5, and 11.5 exhibited clear distinctions to those found in virgin mice (Fig. 1e, f). We then elucidated the comprehensive changes of uterine trNK cells during decidualization by integrating all their upregulated genes in comparison with virgin trNK cells, and subsequently applying these combined genes to GO analysis (Supplementary Fig. 1h, and Fig. 1g, h). Consistent with the aforementioned results, the analysis revealed that the most enriched feature in the uterine trNK cells during decidualization was DNA replication, which is closely associated with cell proliferation. Moreover, we have identified two additional highly enriched features that are closely associated with the effectiveness of NK cells (cytolysis) and the process of apoptosis (lymphocyte apoptotic processes) (Fig. 1h). Next, we assessed the extent of enrichment of these features in uterine trNK cells across the different gestational days. The highest level of DNA replication was observed at gd6.5, as evidenced by the expression of the related genes (Fig. 1i, j). Subsequently, there was a gradual decrease in DNA replication at gd8.5 and gd11.5. Additionally, in line with this trend, we observed an increase in the lymphocyte apoptotic feature at gd11.5 (Fig. 1i, j). Thus, the dynamic changes in these two features were correlated with the changes in the cell number of uterine trNK during the process of decidualization. The effectiveness of uterine trNK cells is crucial for ensuring a successful pregnancy. Interestingly, while the proliferation feature began to decrease at gd8.5, the effector feature reached its peak, as evidenced by the increased expression of *Prf1* and *Gzm*s (Fig. 1i, j). Consistently, the upregulation of *PRF1* and *GZM*s was also observed in activated human decidual NK (dNK) cells during pregnancy^39^. Additionally, *Ifitm*s and *Spp1*, which are associated with uterine trNK activation and function, were also found to be upregulated at gd8.5 (Fig. 1j). The enhanced effectiveness of uterine trNK cells during decidualization was believed to be essential for a successful pregnancy, as they played a crucial role in promoting spiral artery remodeling.

Together, these data suggest a dynamic change in uterine trNK cells during decidualization, characterized by extensive proliferation during the early stage, enhanced effectiveness during the intermediate stage, and the emergence of an apoptotic process during the late stage.

### Single-cell transcriptome uncovers a compositional change of trNK during decidualization

**Fig. 2:**
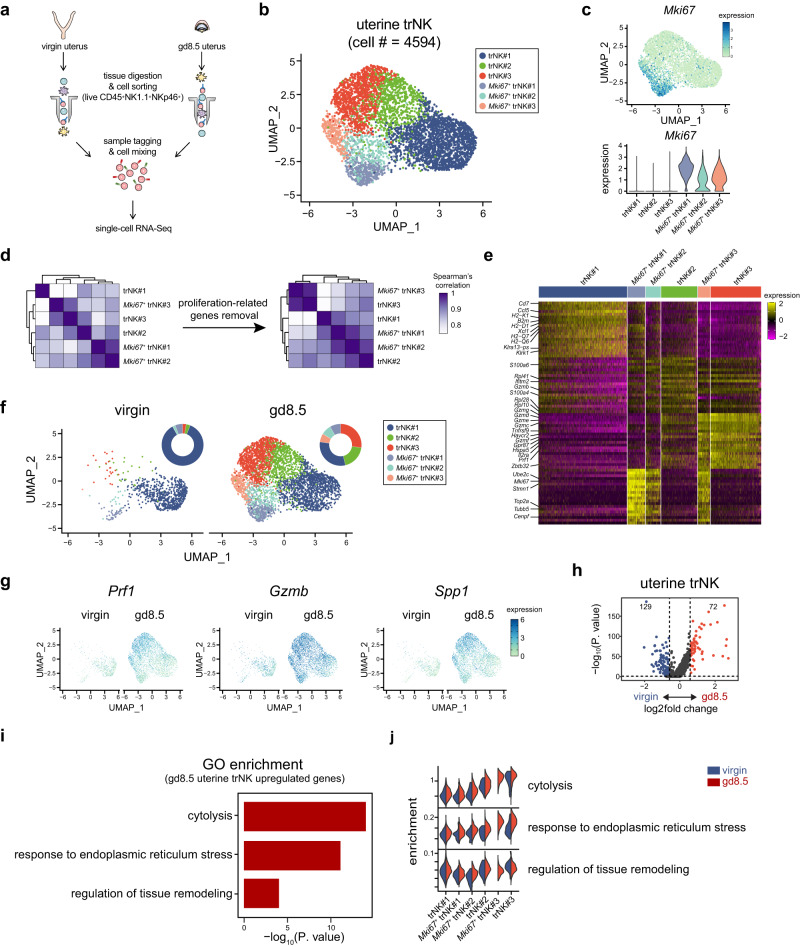
Single-cell transcriptome analysis reveals a compositional change of trNK at gd8.5. **a** Schematic diagram depicting the procedure of scRNA-Seq library preparation for uterine NK/ILC1. Pooled uteri from five virgin mice or two gd8.5 mice are used. NK/ILC1 cells are sort-purified by a gating strategy of live CD45^+^NK1.1^+^NKp46^+^, followed with labeling by different sample tags and mixed together for subsequent scRNA-Seq library preparation. **b** UMAP visualization of uterine trNK from virgin and gd8.5 mice. Colors indicate different uterine trNK subgroups. **c** UMAP of *Mki67* expression in uterine trNK (top), and violin plots of *Mki67* expression in each uterine trNK subgroup (bottom). **d** Spearman’s correlation between each uterine trNK subgroup. Whole transcriptomes (left) or transcriptomes with the exclusion of proliferation-related genes (right) are used for the analysis. **e** Heatmap of the expression of the top 20 specific genes in trNK#1, trNK#2, and trNK#3 subgroups and top 20 proliferation-related genes in the three *Mki67*^+^ trNK subgroups. **f** UMAP visualization of uterine trNK in virgin (left) and gd8.5 (right) mice. Colors indicate different uterine trNK subgroups, and ring pie charts show the proportion of each subgroup. **g** UMAP plot showing expression of *Prf1*, *Gzmb*, and *Spp1* in uterine trNK from virgin and gd8.5 mice. **h** Volcano plot showing differentially expressed genes between uterine trNK from virgin and gd8.5 mice. **i** GO enrichment of the upregulated genes in gd8.5 uterine trNK (**h**). **j** Split violin plots showing module score difference between virgin and gd8.5 uterine trNK in each subgroup. The enriched GO terms in (**i**) was utilized. Data are representative of two independent experiments (**b**–**j**).

The enhancement of uterine trNK efficacy was crucial for ensuring a successful pregnancy. However, the specific regulatory mechanism still remained elusive. Therefore, we focused our examination on the alterations in gd8.5 uterine trNK cells. NK1.1^+^NKp46^+^ uterine NK/ILC1 cells were sort-purified from gd8.5 pregnant mice and virgin mice (Supplementary Fig. 2a). Subsequently, the cells labeled with different sample tags were mixed together and subjected to single-cell RNA sequencing (scRNA-Seq) analysis (Fig. 2a). Following quality control, we obtained a total of 8019 high-quality cells, with 4594 (58.0% of the total) identified as trNK cells (Supplementary Fig. 2b–d). Further, the trNK cells could be classified into six subgroups (Fig. 2b). Three of them exhibited a notable proliferative characteristic, as evidenced by the presence of *Mki67* expression (Fig. 2c and Supplementary Fig. 2e). Nevertheless, when this characteristic was disregarded, these *Mki67*^+^ subgroups were found to bear a resemblance to the remaining *Mki67*^-^ subgroups (Fig. 2d). Consistently, the signature genes of the *Mki67*^-^ subgroups showed comparable expression levels in the corresponding *Mki67*^+^ subgroups (Fig. 2e). Hence, the six trNK subgroups were referred to as trNK#1, trNK#2, trNK#3, *Mki67*^+^ trNK#1, *Mki67*^+^ trNK#2, and *Mki67*^+^ trNK#3 (Fig. 2b).

Next, the uterine trNK from gd8.5 and virgin mice were distinguished based on their respective sample tags (Fig. 2a). Intriguingly, trNK#2, trNK#3, *Mki67*^+^ trNK#2 and *Mki67*^+^ trNK#3 demonstrated a significant prevalence at gd8.5, raising a possibility that they might correspond to the peak effectiveness of trNK at the particular time point (Fig. 2f). In line with this, these subgroups, especially trNK#3 and *Mki67*^+^ trNK#3, showed elevated expression of *Prf1*, *Gzmb* and *Spp1* (Fig. 2g). Then, contribution of this compositional alteration to the trNK transcriptome change at gd8.5 was evaluated. Overall, a total of 72 genes exhibited increased expression at gd8.5 compared to virgin mice (Fig. 2h). They were enriched in biological processes related to ‘cytolysis’, ‘responses to endoplasmic reticulum stress’ and ‘regulation of tissue remodeling’ (Fig. 2i). Then, the enrichment of these features in each trNK subgroups was compared (Fig. 2j). The enrichment of ‘cytolysis’, which reflected the effectiveness of trNK cells, was found to be comparable before and after pregnancy. However, its enrichment showed a gradual increase in the trNK#2, *Mki67*^+^ trNK#2 subgroups and the trNK#3, *Mki67*^+^ trNK#3 subgroups, corroborating that the compositional change corresponded to the enhanced effectiveness of uterine trNK at gd8.5 (Fig. 2j and Supplementary Fig. 2f). The enrichment of the ‘response to endoplasmic reticulum stress’ was significantly increased in trNK#3 and *Mki67*^+^ trNK#3 of gd8.5 mice, but not in virgin mice, indicating that the feature was induced by pregnancy (Fig. 2j and Supplementary Fig. 2g). The enrichment of the ‘regulation of tissue remodeling’ was associated with the reported role of uterine trNK in spiral artery remodeling. This feature was primarily enriched in the three non-proliferative trNK subgroups, indicating that they might play a crucial role in facilitating the tissue remodeling process (Fig. 2j and Supplementary Fig. 2h). Thus, the transcriptomic change in gd8.5 trNK cells could be attributed to both the compositional alterations within different subgroups and the changes in gene expression occurring within each subgroup. In contrast, these changes were nearly negligible in gd8.5 cNK or ILC1 cells, suggesting the presence of a distinct regulatory mechanism specifically governing the alterations in trNK cells (Supplementary Fig. 2i, j).

Collectively, the scRNA-Seq analysis uncovers a previously unrecognized compositional change of uterine trNK cells at gd8.5, which is presumed to primarily contribute to the augmented effectiveness of trNK cells.

### 4-1BB and CD55 denote a differentiation program during trNK activation

**Fig. 3:**
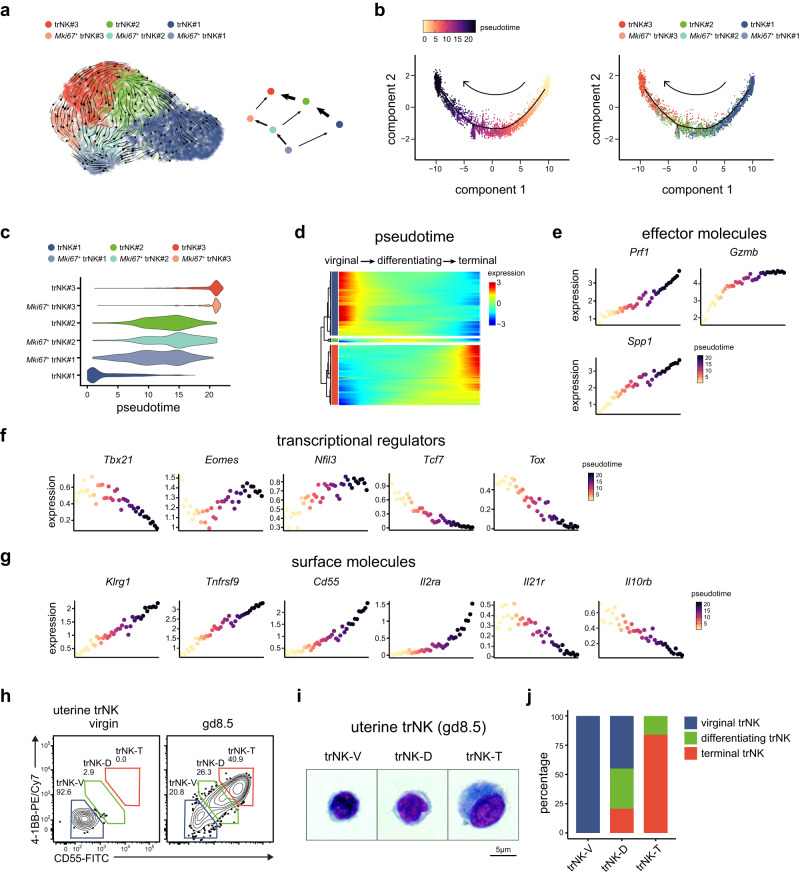
The differentiation of trNK is denoted by 4-1BB and CD55 expression. **a** RNA velocity of the differentiation trajectory of uterine trNK. **b** Pseudotime inference of uterine trNK differentiation in a two-dimensional state-space defined by Monocle2 (left), and ordering of each uterine trNK subgroup along the trajectory (right). **c** Distribution of each uterine trNK subgroup along the pseudotime trajectory. **d** Gene expression dynamics of uterine trNK along pseudotime trajectory. **e** Expression of the indicated effector genes in uterine trNK, respected to pseudotime coordinate. **f** Expression of the indicated transcription factors in uterine trNK, respected to pseudotime coordinate. **g** Expression of the indicated surface molecules in uterine trNK, respected to pseudotime coordinate. **h** Flow cytometry showing co-expression of 4-1BB and CD55 on uterine trNK in virgin and gd8.5 mice. **i** Giemsa-Wright staining showing the morphology of uterine trNK-V, trNK-D and trNK-T subgroups from gd8.5 dams. **j** Deconvolution of the transcriptomes (bulk RNA-Seq) of uterine trNK-V, trNK-D and trNK-T subgroups, using the gene signatures of virginal, differentiating, and terminal trNK subgroups (based on scRNA-Seq). Numbers indicate the percentages in each box. Data are shown as the mean ± SEM. Data are representative of two independent experiments (**a**–**j**) or at least three independent experiments (**h**–**i**). Source data are provided as a Source Data file.

The presence of newly identified trNK subgroups at gd8.5 suggested the likelihood of a trNK differentiation program. Indeed, based on RNA velocity analysis, we uncovered a differentiation trajectory of uterine trNK cells, originating from the trNK#1 subgroup, progressing through the trNK#2 subgroup and ultimately culminating in the trNK#3 subgroup (Fig. 3a). In addition, there was a parallel trajectory among the three *Mki67*^+^ subgroups, with each subgroup demonstrating a propensity to transition into its corresponding *Mki67*^-^ counterpart. Furthermore, a pseudotime analysis was conducted to examine the differentiation of uterine trNK cells, which revealed that the trNK#3 and *Mki67*^+^ trNK#3 subgroups, as well as the trNK#2 and *Mki67*^+^ trNK#2 subgroups, were in the same stages of differentiation (Fig. 3b, c). Whereas, the *Mki67*^+^ trNK#1 subgroup exhibited a more differentiated state compared to the trNK#1 subgroup (Fig. 3c). Nevertheless, due to their presence in virgin mice, they were collectively referred to as virginal trNK (Fig. 2f and Supplementary Fig. 3a). Whereas, the trNK#2 and *Mki67*^+^ trNK#2 subgroups were referred to as differentiating trNK, and the trNK#3 and *Mki67*^+^ trNK#3 subgroups were termed as terminal trNK, based on their differentiation stages (Supplementary Fig. 3a). Along the trNK differentiation trajectory, three gene expression dynamics were identified that corresponded to the differentiation stages (Fig. 3d). Recently, Roser Vento-Tormo et al. had also reported the existence of human decidual NK (dNK) subgroups, which they termed dNK1, dNK2 and dNK3 (Supplementary Fig. 3b)^39^. We found that these human dNK subgroups exhibited a significant similarity in transcriptome with the gd8.5 uterine trNK subgroups in mice (Supplementary Fig. 3c). Furthermore, the human dNK cells also undergo a differentiation progress, transitioning from the dNK3 subgroup to the dNK1 subgroup while passing through the intermediate dNK2 subgroup (Supplementary Fig. 3d, e). According to their report, the dNK1 subgroup, which had demonstrated the highest effectiveness, was supposed to play a critical role in the process of spiral artery remodeling through its interaction with the extravillous trophoblasts (EVTs). Consistently, the expression of NK cell effector molecules *PRF1, GZMB*, *GZMA* and *GNLY* was observed to increase along the trajectory (Supplementary Fig. 3g). The expression dynamics of genes related to trNK function in mice were also assessed along the differentiation trajectory. Similarly, the levels of effector molecules such as *Prf1*, *Gzma*, *Gzmb* and *Spp1* were found to gradually increase (Fig. 3e and Supplementary Fig. 3f). In contrast, as trNK differentiate, there was a decrease in the expression of *Ifng*, but the absolute expression of *Ifng* remained consistently low (Supplementary Fig. 3f). And during the differentiation process, several transcription regulators, including *Eomes*, *Nfil3*, *Runx3*, *Id2* and *Prdm1*, were observed to be upregulated, whereas *Tbx21*, *Tcf7*, *Tox*, *Gata3*, and *Stat1* were downregulated (Fig. 3f and Supplementary Fig. 3h). Taken together, these data suggest that the observed differentiation program may have a general role in mammals to enhanced the effectiveness of uterine trNK cells, which play a crucial role in ensuring a successful pregnancy.

The human dNK subgroups could be distinguished based on their differential expression of CD39, ITGB2, CD103 and KIR2DL1. To identify markers characterizing the uterine trNK subgroups in mice, the differential expression of surface molecules along the trNK differentiation trajectory was profiled. Usually, *Itgam* (encoding CD11b) and *Cd27* were used to indicate the maturation of NK cells^40,41^. Nevertheless, they were both downregulated along the trajectory, suggesting that this differentiation process was distinct from the maturation of NK cells (Supplementary Fig. 3i). Whereas, *Klrg1*, a marker acquired by mature NK cells^41^, was upregulated during the differentiation (Fig. 3g). In addition, other surface molecules, including *Tnfrsf9*, *Cd55*, and *Il2ra*, were also upregulated, while *Il10rb* and *Il21r* were downregulated along the trNK differentiation trajectory (Fig. 3g). The expression of *Tnfrsf9* (encoding 4-1BB) and *Cd55* on NK cells were not reported to be associated with their development or maturation. However, they were differentially expressed in the uterine trNK subgroups, and they cooperatively denoted the differentiation stages of trNK (Supplementary Fig. 3j, k). The expression of 4-1BB and CD55 on uterine trNK was confirmed through flow cytometry (Fig. 3h). Consistently, 4-1BB and CD55 were expressed on uterine trNK cells at gd8.5, but not in virgin mice. Based on their expression levels, the trNK cells were also divided into three subgroups, trNK-V (4-1BB^-/low^CD55^-/low^), trNK-D (4-1BB^int^CD55^int^) and trNK-T (4-1BB^high^CD55^high^), corresponding to the three differentiation stages of trNK (Fig. 3h and Supplementary Fig. 3a). Morphologically, the trNK cells showed a gradual increase in cell size and cytoplasmic granularity as they progressed through the differentiation stages (Fig. 3i and Supplementary Fig. 3l). Furthermore, the correspondence of these three trNK subgroups to the three scRNA-Seq defined trNK differentiation stages was confirmed by subjecting their transcriptomes to deconvolution algorithms (Fig. 3j).

The expansion of uterine trNK during pregnancy is still a subject of debate, particularly regarding whether it is due to local proliferation or cell migration. After an intravenous anti-CD45 antibody injection at gd8.5, we found that none of the uterine trNK cells were labeled, consistent with the idea that they were tissue resident and proliferated locally (Supplementary Fig. 3m). Furthermore, we examined the impact of cell migration inhibition on uterine trNK cells. Through daily treatment with FTY720, we observed a significant reduction in T cells within the uterus at gd8.5, indicating the effectiveness of the migration inhibition (Supplementary Fig. 3n). Nevertheless, the number of NK/ILC1, as well as the differentiation of trNK, remained unaffected (Supplementary Fig. 3n, o).

Collectively, these data suggest that there is a differentiation of the uterine-localized trNK cells during pregnancy, which substantially enhances their effectiveness.

### The initiation of trNK differentiation is promoted by an IL-21R-STAT3 axis

**Fig. 4:**
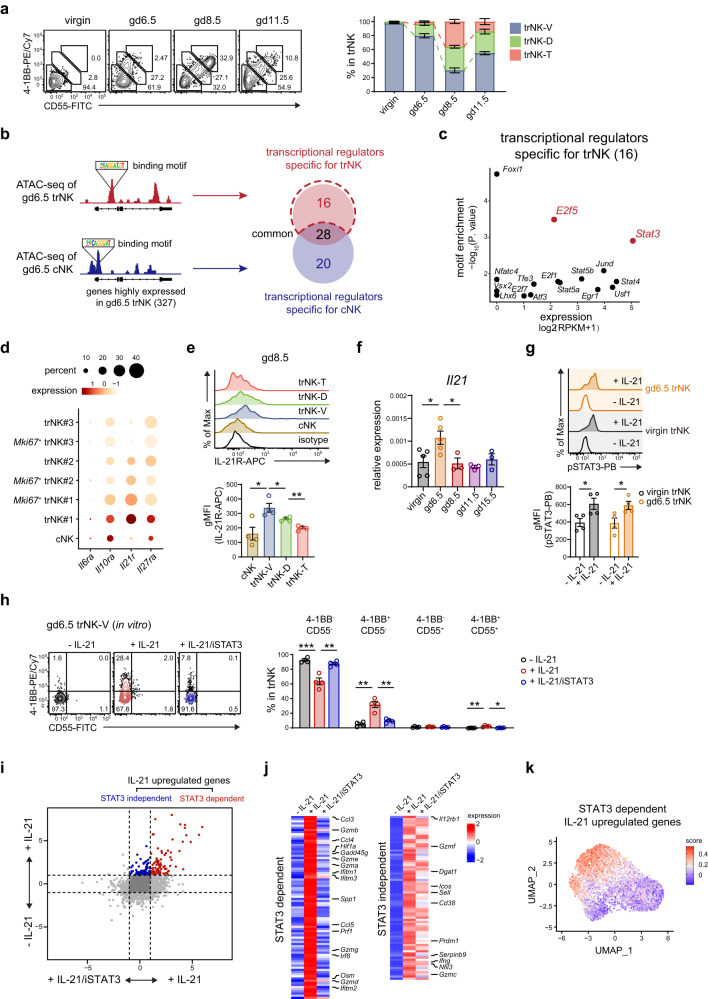
IL-21R-STAT3 axis promotes initial differentiation of trNK. **a** Flow cytometry and the corresponding statistics showing compositional change of uterine NK in virgin mice and pregnant mice at the indicated gestational days (*n* = 3, 4, 4, and 3 per group). **b** Schematics of prediction for potential transcription regulators (related to Supplementary Fig. 4a). **c** Scatter plots showing the motif enrichment and expression of potential trNK-specific transcription regulators. **d** Dot plot showing the expression of cytokine receptors associated with STAT3 activation. **e** Flow cytometric histogram showing IL-21R expression on uterine trNK-V, trNK-D, trNK-T subgroups and cNK from gd8.5 mice (*n* = 4 per group; *P = 0.0152, *P = 0.0483, **P = 0.0011). **f** Quantitative PCR assessing *Il21* expression in uteri at the indicated gestational days (*n* = 5, 5, 3, 3, and 3 per group; *P = 0.0258, *P = 0.0377). **g** Flow cytometric and statistical analysis of STAT3 phosphorylation in uterine trNK. Uterine trNK are sorted from virgin and gd6.5 mice, and stimulated in presence or absence of recombinant IL-21 for 30 min (*n* = 4 per group; *P = 0.0348, *P = 0.0316). (**h**-**k**) Uterine trNK-V subgroup is sorted from gd6.5 mice, and cultured in presence or absence of IL-21 and STAT3 inhibitor (iSTAT3) for 72 h. **h** Flow cytometric and statistical analysis showing the percentage of 4-1BB^-^CD55^-^, 4-1BB^+^CD55^-^, 4-1BB^-^CD55^+^ and 4-1BB^+^CD55^+^ trNK subgroups (*n* = 4 per group; ***P = 0.0010, **P = 0.0019, **P = 0.0011, **P = 0.0021, **P = 0.0078, *P = 0.0122). **i** Dot plot depicting the gene expression changes of in vitro cultured uterine trNK (**h**). **j** Heatmap showing expression change of the STAT3-dependent and -independent genes (**i**) **k** Scoring of the STAT3-dependent IL-21-upregulated genes in gd8.5 uterine trNK. Numbers indicate the percentages in each box. Data are shown as the mean ± SEM. *P*-values are calculated by two-sided unpaired *t*-test, *P < 0.05, **P < 0.01, ***P < 0.001. Data are representative of two independent experiments (**b**–**d**, **i**–**k**) or at least three independent experiments (**a**, **e**–**h**). Source data are provided as a Source Data file.

Although the regulation of uterine trNK function during pregnancy had been previously reported^26^, the newly discovered differentiation program suggested the existence of a distinct regulatory mechanism. Based on a tracing of the 4-1BB and CD55 expression on uterine trNK cells during gestation, we observed that the trNK differentiation commenced at gd6.5 (Fig. 4a). We further identified 400 upregulated genes in trNK cells at gd6.5 compared to virgin mice (Supplementary Fig. 1h). Notably, 327 of them showed low expression in gd6.5 cNK, indicating their potential involvement in the trNK differentiation (Supplementary Fig. 4a). To unravel the mechanism underlying the upregulation of these genes, we performed an assay for transposase-accessible chromatin with high-throughput sequencing (ATAC-seq) analysis, which enabled the identification of chromatin regions that were accessible and potentially involved in gene regulation. Subsequently, potential transcriptional regulators for the 327 genes in gd6.5 trNK and cNK cells were predicted, based on the presence of binding motifs associated with these transcriptional regulators within the relevant accessible chromatin regions (Fig. 4b). The specific regulators for trNK differentiation were filtered based on the assumption that they should preferentially function in trNK cells and exhibit substantial expression levels (Fig. 4b, c). Finally, we identified two transcription factors E2F5 and STAT3 (Fig. 4c). E2F5 was commonly associated with cell cycle arrest and functioned as a transcriptional repressor. Therefore, we pursued further investigation on STAT3, a critical transcriptional regulator also known for its involvement in Th17 differentiation. The regulatory role of STAT3 was activated by upstream cytokines, including IL-6, IL-21, IL-23 and IL-27^27,42^. In line with this, *Il21r* exhibited particularly high expression in the trNK#1 subgroup, while also being substantially expressed in the *Mki67*^+^ trNK#1, trNK#2 and *Mki67*^+^ trNK#2 subgroups (Fig. 4d). In contrast, its expression in the trNK#3 and *Mki67*^+^ trNK#3 subgroups, as well as cNK cells, was significantly lower. This differential expression of IL-21R among the trNK subgroups characterized by 4-1BB and CD55, as well as in cNK cells, was also confirmed by flow cytometry (Fig. 4e, and Supplementary Fig. 4b). In addition, we observed that *Il21* exhibited the highest expression in the uterus at gd6.5 (Fig. 4f). Macrophages displayed significant *Il21* expression, while T cells exhibited mild *Il21* expression but had a pronounced expansion at gd6.5, suggesting that these cells might serve as the primary sources of IL-21 (Supplementary Fig. 4c). Moreover, stimulation of IL-21 resulted in the phosphorylation of STAT3 in trNK cells from both gd6.5 and virgin mice (Fig. 4g). Together, these findings suggested the potential involvement of the IL-21R-STAT3 axis in the initiation of uterine trNK differentiation.

Next, we further verified this possibility. Firstly, whether IL-21 could directly act on trNK cells to initiate the differentiation program was assessed. Thus, the trNK-V subgroup from gd6.5 mice were sort-purified and subsequently cultured in vitro in the absence or presence of recombinant murine IL-21. And, a STAT3 inhibitor (iSTAT3) was utilized to further elucidate the potential involvement of STAT3 in this process. After 72 h, we observed that IL-21 stimulation resulted in an increase in the cell size and cytoplasmic granularity of trNK cells, as demonstrated by flow cytometry (Supplementary Fig. 4d). However, these changes were reversed upon iSTAT3 treatment. Additionally, staining for 4-1BB and CD55 demonstrated that IL-21 stimulation promoted the upregulation of 4-1BB, but not CD55, suggesting that CD55 upregulation might be regulated by a separate pathway (Fig. 4h). Notably, iSTAT3 treatment effectively inhibited the upregulation of 4-1BB, providing further support for the involvement of the STAT3 pathway in this process (Fig. 4h). To comprehensively explore the changes in trNK cells following IL-21 stimulation, we performed an RNA-Seq analysis on these in vitro cultured trNK (Fig. 4i). Then, the IL-21 upregulated genes were categorized into those dependent on STAT3 and those independent of STAT3 (Fig. 4j). Multiple effector molecules, including *Prf1*, *Gzma*, *Gzmb* and *Spp1*, were found to be upregulated by IL-21 through the activation of STAT3 (Fig. 4j). Furthermore, during trNK cell differentiation, there was an observed increase in the expression of these IL-21 upregulated genes that were dependent on STAT3 (Fig. 4k). Collectively, our results demonstrated that the IL-21R-STAT3 axis played a critical role in promoting the differentiation of uterine trNK cells during decidualization.

In addition, IL-21 had also been reported to direct an prompt NK cell activation response. To ensure the NK cell reactivity, chromatin regions of their effector genes were usually already accessible, eliminating the need for alteration during activation. Conversely, the differentiation of immune cells often demanded a substantial amount of time to complete chromatin accessibility changes. For instance, the in vitro differentiation process of CD4^+^ T helper (Th) cells typically spanned ~72 h. As a result, the transcriptome and chromatin accessibility of differentiated Th cells underwent notable changes in comparison to naïve T cells. The similar transcriptome changes were also observed in uterine trNK cells during decidualization (Supplementary Fig. 1h). We further characterized the impact of prolonged IL-21 stimulation on in vitro cultured uterine trNK cells. The trNK-V subgroup sort-purified from gd6.5 mice was cultured in the absence or presence of recombinant murine IL-21. Subsequently, RNA-Seq analysis was conducted at 0, 6, 24 and 72-h time points (Supplementary Fig. 4e). By comparing the trNK transcriptomes in the absence and presence of IL-21 at different time points, we found that prolonged IL-21 stimulation induced a substantial transcriptome change, corroborating that the trNK cells were undergoing differentiation (Supplementary Fig. 4f). At 72 h, we also conducted an assessment of the chromatin accessibility of trNK cells using ATAC-Seq (Supplementary Fig. 4g). Consistently, the prolonged stimulation of IL-21 induced substantial changes in chromatin accessibility in trNK cells (Supplementary Fig. 4h). In particular, we examined the changes at the *Spp1*, *Tnfrsf9* and *Klrg1* loci, and observed an increase in the accessibility of their open chromatin regions (OCRs) (Supplementary Fig. 4i). Altogether, these results further confirm that prolonged IL-21 stimulation directly promotes the differentiation of trNK cells.

### *Il21r* deficiency substantially affects the trNK differentiation during decidualization

**Fig. 5:**
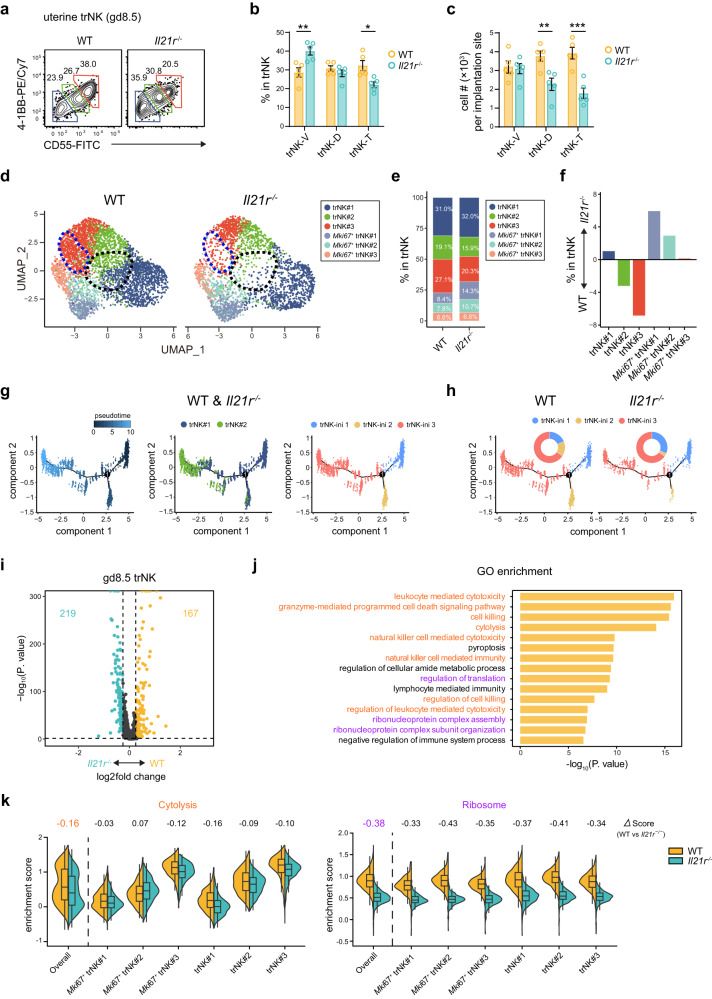
*Il21r* deficiency diminishes trNK differentiation during decidualization. **a** Flow cytometry showing uterine trNK differentiation in gd8.5 WT and *Il21r*^-/-^ dams. **b** Percentages of uterine trNK subgroups in gd8.5 WT and *Il21r*^-/-^ dams (*n* = 5 per group; **P = 0.0083, *P = 0.0111). **c** Cell number of each uterine trNK subgroup in gd8.5 WT and *Il21r*^-/-^ dams at one implantation site (*n* = 5 per group; **P = 0.0076, ***P = 0.0009). **d** UMAP of uterine trNK from gd8.5 WT and *Il21r*^-/-^ dams. Colors indicate different uterine trNK subgroups, ring pie charts show distribution of each subgroup, and dashed-line circles indicate uterine trNK deficits in *Il21r*^-/-^ mice. **e** Percentage of each uterine trNK cluster in gd8.5 WT and *Il21r*^-/-^ dams. **f** Proportion change of each uterine trNK subgroup between gd8.5 WT and *Il21r*^-/-^ dams. **g** Pseudotime analysis of trNK#1 and trNK#2 (left) and ordering of the two subgroups along the trajectory (middle). Accordingly, these cells are divided into three refined subgroups. **h** Distribution of the refined trNK subgroups (**f**) in WT and *Il21r*^-/-^ dams. Ring pie charts show the proportion of each subgroup. **i** Volcano plots depicting differentially expressed genes (log_2_ fold change > 0.25 and *P*-value < 0.05) between uterine trNK from gd8.5 WT and *Il21r*^-/-^ dams. **j** GO enrichment of the upregulated genes in uterine trNK of WT versus *Il21r*^-/-^ dams. **k** Split violin plots showing module scores of cytotoxicity-associated and ribosome-associated features in total trNK cells and each individual uterine trNK cluster from WT and *Il21r*^-/-^ dams (*n* = 3816, 2733, 321, 392, 296, 292, 252, 185, 1181, 874, 730, 435, 1036, and 555 per group). Each box represents the interquartile range (IQR), horizontal line inside represents the median, and extend whiskers indicate 1.5 times of the IQR. Numbers indicate the percentages in each box. Data are shown as the mean ± SEM. *P*-values are calculated by two-sided unpaired *t*-test, *P < 0.05, **P < 0.01, ***P < 0.001. Data are representative of at least three independent experiments (**a**–**c**) or two independent experiments (**d**–**i**). Source data are provided as a Source Data file.

We further demonstrated the essential role of IL-21 signaling in the in vivo differentiation of trNK cells. We observed substantial expression of IL-21R on NK/ILC1 cells, as well as mild expression on T and B cells within the uterus (Supplementary Fig. 5a). NK/ILC1 were well-known as the predominant immune population at the maternal-fetal interface, and the expansion of trNK cells during decidualization was vital for spiral artery remodeling. According to our hypothesis that the IL-21R-STAT3 axis promoted uterine trNK differentiation, this process would be impaired in *Il21r*^-/-^ mice. Indeed, while the number of uterine trNK cells was unaffected in virgin mice (Supplementary Fig. 5b), a significant reduction was observed in gd8.5 *Il21r*^-/-^ dams (Supplementary Fig. 5c). In contrast, the numbers of cNK, ILC1, as well as T cells, B cells, DC and macrophages remained unaltered both before and after pregnancy (Supplementary Fig. 5b-d). Further, the differentiation of uterine trNK cells at gd8.5 was examined based on the expression of 4-1BB and CD55 (Fig. 5a). As anticipated, changes in the distribution of the three trNK subgroups were observed, with a notable reduction in the trNK-D and trNK-T subgroups (Fig. 5b, c). Hence, the deficiency of *Il21r* disrupted the differentiation of trNK cells during decidualization. To further elucidate the impairment of uterine trNK cell differentiation in *Il21r*^-/-^ dams, a scRNA-Seq analysis was conducted on gd8.5 trNK cells isolated from both WT and *Il21r*^-/-^ dams (Fig. 5d). Consistently, we observed a clear deficiency of trNK cells at both the initial and terminal differentiation stages in *Il21r*^-/-^ dams. Particularly, the changes in distribution of the six scRNA-Seq defined trNK subgroups (trNK#1, trNK#2, trNK#3, *Mki67*^+^ trNK#1, *Mki67*^+^ trNK#2, and *Mki67*^+^ trNK#3) in the *Il21r*^-/-^ dams were examined (Fig. 5e). The most notable reductions were found in the trNK#2 and trNK#3 subgroups, corroborating that the deficiency of *Il21r* affected both the initial and terminal stages of uterine trNK cell differentiation (Fig. 5f).

We just showed that IL-21 stimulation directly promoted the initial differentiation of trNK in vitro. Thus, the deficiency of the trNK#2 subgroup in *Il21r*^-/-^ uterine trNK cells at gd8.5 was firstly explored. To interpret their impairment during the initial differentiation process, we conducted a detailed analysis of the pseudotime trajectory from trNK#1 to trNK#2 (Fig. 5g). Based on the distribution of cells along the trajectory, we further classified the trNK cells into three distinct stages, trNK-ini 1, trNK-ini 2 and trNK-ini 3. A significant impairment of trNK-ini 2 was observed in *Il21r*^-/-^ gd8.5 dams, corroborating that the absence of IL-21 signal adversely affected the differentiation of uterine trNK cells (Fig. 5g, h). Next, an overall transcriptome comparison between WT and *Il21r*^-/-^ gd8.5 uterine trNK cells was performed (Fig. 5i and Supplementary Data). GO analysis revealed that the IL-21 signal primarily promoted cytotoxicity-associated and ribosome-associated features in gd8.5 uterine trNK cells (Fig. 5j). Further, the differences in gene expression between each cluster of uterine trNK cells in gd8.5 WT and *Il21r*^-/-^ dams were compared (Supplementary Fig. 5e and Supplementary Data). While each trNK cluster exhibit a moderate decrease in cytotoxicity-associated features in *Il21r*^-/-^ mice, the overall transcriptome comparison revealed a substantially larger reduction, indicating that the overall change might also be partly attributed to altered cell composition, stemming from the differentiation defect of *Il21r*^-/-^ uterine trNK cells (Fig. 5d,k). On the other hand, we observed a consistent reduction in ribosome-associated features across all subgroups of uterine trNK cells in *Il21r*^-/-^ dams, suggesting that they were direct downstream targets of the IL-21 signal (Fig. k, and Supplementary Fig. 5f, g). Together, our results confirm the requirement of the IL-21 signal in initiating the differentiation of uterine trNK cells during decidualization.

### *Il21r* deficiency leads to increased incidence of miscarriage and impaired spiral artery remodeling

**Fig. 6:**
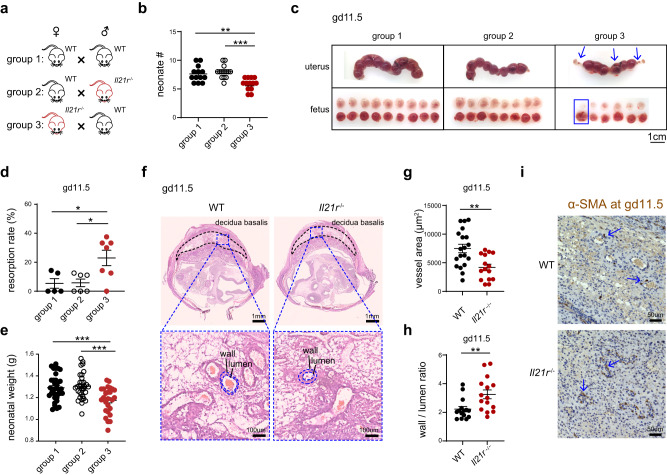
*Il21r* deficiency leads to increased incidence of miscarriage and defective spiral artery remodeling. **a** Mating strategy for experiments in (**b**–**e**) and (Supplementary Fig. 6a-e). **b** Numbers of neonates from groups 1, 2, and 3 (*n* = 13, 14, and 13 per group; **P = 0.0013, ***P < 0.0001). **c** Implanted embryos in uteri of gd11.5 dams from groups 1, 2, and 3. Blue arrow and rectangle indicate resorbed fetus. **d** Resorption rate in gd11.5 dams from groups 1, 2, and 3 (*n* = 5, 6, and 7 per group; *P = 0.0293, *P = 0.0188), calculated by (the number of resorptions) / (the number of resorptions and normal implantations). **e** Body weight of neonates from groups 1, 2, and 3 on the day of birth (*n* = 35, 33, and 26 per group; ***P = 0.0004, ***P = 0.0001). **f** Hematoxylin and Eosin (H & E) staining showing the whole implantation site from gd11.5 WT (left) and *Il21r*^-/-^ (right) dams (top). Remodeling of spiral arteries in decidua basalis are assessed by their wall and lumen area (bottom). **g** Quantification of vessel wall areas of spiral arteries from gd11.5 WT and *Il21r*^-/-^ dams (*n* = 19 and 15 per group; **P = 0.0018). Each data point represents the mean of five measurements in an implantation site. **h** Quantification of the ratio between vessel wall area and the corresponding lumen area in spiral arteries of gd11.5 WT and *Il21r*^-/-^ dams (*n* = 15 per group; **P = 0.0075). Each data point represents the mean of five measurements in an implantation site. **i** Immunohistochemical analysis of smooth muscle actin (SMA, brown) in gd11.5 WT and *Il21r*^-/-^ dams. Blue arrows indicate the increased thickness of spiral artery vessel walls in *Il21r*^-/-^ dams. Scale bar, 50 μm. Data are shown as the mean ± SEM. *P*-values are calculated by two-sided unpaired *t*-test, *P < 0.05, **P < 0.01, ***P < 0.001. Data are representative of at least three independent experiments (**b**–**i**). Source data are provided as a Source Data file.

Uterine trNK played a crucial role in facilitating the process of spiral artery remodeling during decidualization^43^. We found that the NK cells presented in the decidua were primarily the differentiated trNK subset, distinguished by their expression of CD55 (Supplementary Fig. 6a). In contrast, the majority of NK cells in the myometrium lacked CD55 expression. It raised a possibility that the differentiated trNK cells might be specifically essential for the process of spiral artery remodeling. Thus, we wondered whether the impaired differentiation of trNK cells in *Il21r*^-/-^ dams could hinder the successful progression of gestation. Indeed, newborns from *Il21r*^-/-^ dams exhibited a notable decrease in numbers. To verify whether it was attributed to the maternal *Il21r* deficiency, a comparison was conducted among three mating groups: WT female mated with WT male, WT female mated with *Il21r*^-/-^ male, and *Il21r*^-/-^ female mated with WT male (Fig. 6a). The result showed a significant reduction in the number of neonates from the third mating group, demonstrating that the maternal *Il21r* deficiency had indeed led to pregnancy complications (Fig. 6b). Further, the change in fetuses number was examined. At gd6.5, the fetus numbers were relatively similar among the three mating groups, ruling out the possibility of implantation failure (Supplementary Fig. 6b, c). However, at gd11.5, there was a noticeable increase in fetal resorption in the *Il21r*^-/-^ dams of the third group (Fig. 6c, d). Subsequently, at gd15.5, when fetal resorption was more apparent, the increment in the *Il21r*^-/-^ dams was further validated (Supplementary Fig. 6d, e). Additionally, we observed reduced body weight in neonates from the third mating group, indicating fetal growth restriction (Fig. 6e).

Defective remodeling of spiral arteries had been frequently associated with pregnancy complications, including fetal growth restriction and miscarriage^44–47^. Given the potential involvement of differentiated uterine trNK cells in spiral artery remodeling, we hypothesized that the abnormal differentiation of trNK cells in *Il21r*^-/-^ dams could contribute to defective pregnancies. Thus, we conducted a comparison of spiral artery remodeling between WT and *Il21r*^-/-^ dams at gd11.5, a stage by which the remodeling process was anticipated to be completed^15^. The H&E staining showed almost identical anatomical structures of placentas in both mice (Fig. 6f). However, there was a significant reduction in vessel size in decidua basalis of *Il21r*^-/-^ dams (Fig. 6f, g). In WT dams, the thickness of the vessel wall, indicated by the ratio between vessel and lumen areas, was noticeably decreased at gd11.5, demonstrating normal spiral artery remodeling involving muscular wall disruption and endothelium replacement^21^. In contrast, the vessel walls in *Il21r*^-/-^ dams remained thick, suggesting a defect in spiral artery remodeling (Fig. 6h). This defect was further confirmed by smooth muscle actin staining (Fig. 6i). Additionally, the defective spiral artery remodeling was already observed when uterine trNK differentiation reached its peak at gd8.5 (Supplementary Fig. 6f–h). Together, these findings suggest that spiral artery remodeling, facilitated by uterine trNK cells, is aberrant in *Il21r*^-/-^ dams.

### Caspase 3 inhibition restores spiral artery remodeling in *Il21*^-/-^ dams

As abovementioned, the terminal differentiation stage of uterine trNK was also affected in *Il21r*^-/-^ dams (Fig. 5a-e). However, it was unlikely due to defects in proliferation or activation, as indicated by Ki-67 staining and forward and side scatters on flow cytometry (Supplementary Fig. 7a, b). We observed an enriched apoptotic feature in uterine trNK during the late stage of decidualization (Fig. 1i). In line with it, the terminally differentiated trNK also exhibited increased apoptosis, as evidenced by the upregulation of pro-apoptotic molecules *Casp3* and *Bid*, as well as the downregulation of the anti-apoptotic molecule *Bcl2* (Supplementary Fig. 7c)^48^. Further, caspase 3 activity also increased along with uterine trNK differentiation (Supplementary Fig. 7d). These findings suggested that an enhanced apoptotic process occurred during the differentiation of uterine trNK, which might be necessary to prevent overactivation and subsequent pregnancy failure.

**Fig. 7:**
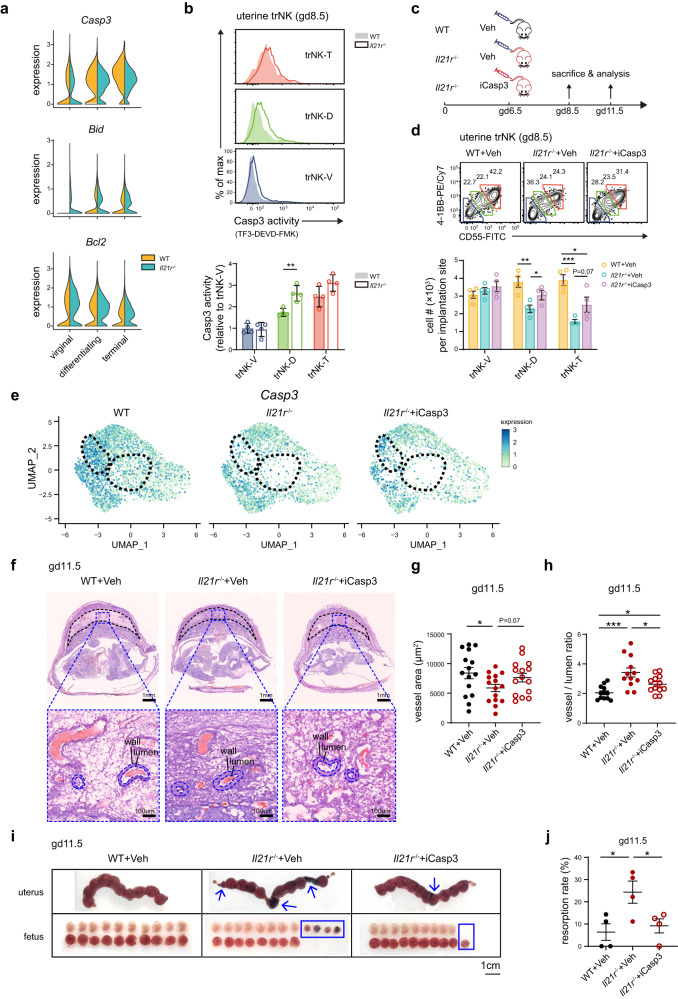
Caspase 3 inhibition recovers spiral artery remodeling in *Il21r*^-/-^ dams. **a** Split violin plots showing expression of pro- (*Casp3* and *Bid*) and anti-apoptotic (*Bcl2*) genes in WT and *Il21r*^-/-^ uterine trNK subgroups (5d). **b** Flow cytometric and statistical analysis of caspase 3 activities in WT and *Il21r*^-/-^ uterine trNK subgroups (5a) (*n* = 4 per group; **P = 0.0049). **c**–**j** Caspase 3 inhibition. **c** Schematics of caspase 3 inhibitor (iCasp3) or vehicle (Veh) administration. **d** Flow cytometric and statistical analysis of each gd8.5 uterine trNK subgroup (*n* = 4 per group; **P = 0.0063, *P = 0.0260, ***P = 0.0004, *P = 0.0379). **e** UMAP of *Casp3* expression in gd8.5 uterine trNK. Each sample represents a pool of five (WT) or two (*Il21r*^-/-^, *Il21r*^-/-^+iCasp3) uteri. **f** H&E staining showing implantation sites in gd11.5 dams with the indicated genotypes and treatments. Dashed black lines indicate decidua basalis. Spiral artery remodeling is assessed by the ratio between vessel wall and lumen area. **g** Vessel wall areas of spiral arteries in gd11.5 dams with the indicated genotypes and treatments. **h** Quantification of spiral artery remodeling in gd11.5 dams with the indicated genotypes and treatments. **g**, **h** Each data point represents mean of five measurements in an implantation site (**g**, *n* = 15 per group; *P = 0.0355; **h**, *n* = 12 per group; ***P = 0.0004, *P = 0.0134, *P = 0.0323). **i** Embryos in uteri of gd11.5 dams with the indicated genotypes and treatments. Blue arrows and rectangles indicate resorbed fetus. **j** Resorption rates in gd11.5 dams with the indicated genotypes and treatments (*n* = 4 per group), calculated by (number of resorptions) /(number of resorptions + successful implantations) (*n* = 4 per group; *P = 0.0280, *P = 0.0432). Numbers indicate the percentages in each box. Data are shown as the mean ± SEM. *P*-values are calculated by two-sided unpaired *t*-test, *P < 0.05, **P < 0.01, ***P < 0.001. Data are representative of two independent experiments (**a**, **e**) or at least three independent experiments (b, d, and f-j). Source data are provided as a Source Data file.

Next, the expression of apoptotic molecules in the uterine trNK of *Il21r*^-/-^ dams was examined (Fig. 7a and Supplementary Table 1). The *Casp3* levels were significantly reduced in the terminal trNK subgroup. Nonetheless, there was an increase in caspase 3 activity, particularly in the trNK-D subgroup of *Il21r*^-/-^ dams (Fig. 7b). Considering the removal of dead cells during scRNA-Seq and flow cytometry analyses, these results suggested that the apoptotic process in differentiated *Il21r*^-/-^ trNK was intensified, resulting in the survival of only terminally differentiated *Il21r*^-/-^ trNK with lower *Casp3* levels. In line with it, the caspase 3 inhibitor was found to be effective in partially restoring the deficit in cell number of the terminally differentiated uterine trNK in *Il21r*^-/-^ dams (Fig. 7c, d). Further, scRNA-Seq indicated that the caspase 3 inhibition primarily addressed the defect in *Il21r*^-/-^ uterine trNK at the terminal differentiation stage (Fig. 7e). In contrast, the decrease in uterine trNK at the early differentiation stage was not rescued, corroborating that it was primarily a differentiation defect (Fig. 7e). Therefore, the IL-21R signal also plays a role in extending the survival of differentiated uterine trNK at the terminal stage.

As abovementioned, the uterine trNK differentiation was crucial in spiral artery remodeling. We further wondered whether restoring the terminally differentiated uterine trNK could improve the defect in spiral artery remodeling observed in *Il21r*^-/-^ dams. Remarkably, inhibition of caspase 3 activity led to the restoration of spiral artery remodeling at gd11.5 in *Il21r*^-/-^ dams, as evidenced by a decrease in the ratio between vessel and lumen area (Fig. 7f-h). In line with it, there was also a reduction in fetal resorption in *Il21r*^-/-^ mice (Fig. 7i, j). Thus, by impeding the apoptosis process in terminally differentiated uterine trNK through caspase 3 inhibition, we successfully rescued the impaired spiral artery remodeling in *Il21r*^-/-^ dams.

## Discussion

The discovery of the trNK population has greatly expanded our understanding of local immunity in peripheral tissues. As innate lymphocytes, they display rapid responsiveness to inflammatory stimuli originating from their specialized tissues. However, given their tissue-residency, it is also crucial to carefully regulate their activation under normal physiological conditions to prevent unintended tissue damage. The mechanisms that govern the regulation of these distinct activation modes are still largely unknown. In our study of the uterine trNK activation during gestation, we have revealed a previously undisclosed differentiation program that greatly enhances their effectiveness. Additionally, we have identified an apoptotic process that acts as a safeguard, preventing excessive activation of terminally differentiated trNK cells. Thus, these findings provide insights into the field of local immunity. It would also be fascinating to further explore whether similar differentiation programs exist in other tissue-resident innate immune cells to ensure their efficient yet safe activation.

The effector role of uterine trNK is crucial for successful gestation^15,43^. During the process, uterus undergoes significant environmental changes, resulting in notable alterations in the proliferation and distribution of uterine trNK cells^3^. In our study, we specifically examined the transcriptome profiles of uterine trNK cells at different gestational days during decidualization. Our findings demonstrate that the proliferative activity of uterine trNK cells is highest in the early stage, but gradually decreased, coinciding with the occurrence of apoptosis. Together, these changes provide insight into the fluctuations in the number of uterine trNK throughout decidualization. In addition, we have also identified a dynamic expression of genes related to NK cell effector function. Interestingly, despite the reduced proliferative activity after gd6.5, the effector activity continues to increase until gd8.5. Our results indicate that this increment is primarily attributed to the differentiation of uterine trNK cells at gd8.5.

To elucidate the activation features of uterine trNK cells, a single-cell transcriptome analysis is conducted on uterine trNK from virgin mice and mice at gd8.5. We observe a clear differentiation pattern among trNK cells in gd8.5 dams. Based on their transcriptome profiles, uterine trNK can be categorized into three subgroups, virginal trNK, differentiating trNK and terminal trNK, representing different stages of differentiation. Notably, the terminal trNK exhibit significant changes in gene expression when compared to virginal trNK, including an upregulation of NK effector genes such as *Prf1* and *Gzm*s. On the other hand, the differentiating trNK display a transitional state, expressing genes that are specific to either virginal trNK or terminal trNK, albeit to a milder extent. Uterine trNK cells in each stage of differentiation consist of two subgroups, a proliferative subgroup and a non-proliferative subgroup. Considering the decreased proliferation of uterine trNK cells after gd6.5, we speculate that the differentiation process in the non-proliferative fraction is of greater importance in facilitating the overall enhancement of effector function in trNK cells at gd8.5, though further evidence is still required. During the adaptive immunity response, the differentiation processes occur more frequently, particularly in CD4^+^ T helper cells (Th)^49^. However, it is still uncommon during the activation of innate immune cells. As a result of the differentiation program of uterine trNK, their effector function is significantly enhanced to facilitate the remodeling of spiral arteries during decidualization. Another similarity between the differentiations of trNK and CD4^+^ Th cells is that they both undergo an apoptotic process at the terminal stage. This process in the terminally differentiated trNK may be necessary in order to prevent tissue damage caused by their overactivation. It partially explains the decrease in uterine trNK at gd15.5. Overall, these processes of differentiation and apoptosis ensure a robust yet safe activation of uterine trNK cells during decidualization.

In a recent study conducted by Roser Vento-Tormo et al., cell type profiling in the human maternal-fetal interface revealed the classification of decidual NK (dNK) cells into three subsets, dNK1, dNK2, and dNK3. These subsets demonstrated a gradual increase in the levels of *PRF1*, *GZMA*, *GZMB* and *GNLY* as they progressed from dNK3 to dNK1^39^. Thus, the findings from our study in mice correspond with these observations in humans, suggesting the presence of similar trNK differentiation programs in both species. Additionally, the study in humans revealed that the highly effective dNK1 cells were believed to interact with EVTs to facilitate spiral artery remodeling. Similarly, our study highlights the significance of uterine trNK cell differentiation towards the terminal stage for remodeling spiral arteries.

In our study, we have also identified two markers, 4-1BB and CD55, that indicate the differentiation program of uterine trNK cells (Fig. 3h–j). 4-1BB is a well-known activation-induced T cell costimulatory molecule^50^. Its signal plays a role in promoting cell survival, proliferation, cytokine production, and preventing activation-induced cell death^50^. On the other hand, CD55 serves as a decay accelerating factor for complement, protecting cells from complement-mediated cytotoxicity^51^. The expression of these markers reliably represents the uterine trNK differentiation process. However, further exploration is needed to determine whether they can indicate trNK differentiation in other tissues. In addition to 4-1BB and CD55, we have also examined other markers, such as *Cd11b*, *Cd27*, and *Klrg1*^40,41^. Although the expression of *Klrg1* does increase along with trNK differentiation, the expression of *Cd11b* and *Cd27* remains negligible in differentiated trNK, indicating that the differentiation process of trNK cells is distinct from the maturation process observed in cNK cells and thus is probably specific to trNK cells. We have also examined the expression of 4-1BB and CD55 at different gestational days. Consistent with changes observed in the transcriptome, we found that the percentage of differentiated trNK cells in the uterus reaches highest level around gd8.5 (Fig. 4a), suggesting that the differentiation process is limited to a short period. Similarly, the upregulation of *Il21* in the uterus is only significant around gd6.5 (Fig. 4f). These results imply that while the differentiation program significantly enhances the effector of trNK cells, it must be tightly restrained during decidualization.

We found that the IL-21R-STAT3 axis is particularly critical in initiating the differentiation program of trNK during decidualization (Fig. 4h and Fig. 5d). IL-21 has been reported to stimulate cNK activation^52,53^. Its receptor is highly expressed in uterine trNK cells, suggesting the importance of IL-21 signaling in trNK cells. In vitro stimulation experiments reveal that IL-21 is sufficient to induce phosphorylation of STAT3 in trNK cells. In contrast, though IL-15 is capable of activating most NK cell populations, and uterine trNK are found to be absent in *Il15*^-/-^ mice^26^, IL-15 stimulation alone does not induce trNK cell differentiation in vitro. However, when supplemented with IL-21 stimulation, the differentiation process becomes evident. In addition, we find that deficiency in *Il21r* disrupts the initial differentiation of uterine trNK, leading to impaired spiral artery remodeling, fetal growth restriction and fetal resorption. Thus, the IL-21 signal plays a particularly important role in the differentiation of trNK during decidualization. Interestingly, the expression of IL-21R is reduced in differentiated trNK, which may also serve as a mechanism to prevent overactivation. It remains unclear whether the differentiation of trNK cells occurs during an anti-infectious response, but the expression of IL-21 in the tissue environment or the activation of STAT3 is likely an important prerequisite.

We have also observed a decrease in ribosome-associated features in *Il21r*^-/-^ uterine trNK cells, indicating that these features are regulated by the IL-21R-STAT3 axis (Fig. 5i, j). It has been shown that ribosome activity is closely linked to the activation and effector function of immune cells^54^. Thus, it is possible that ribosome-activity is associated with the cytokine secretory activity that characterizes differentiated trNK cells. In line with it, we have also noticed an increase in ER stress-associated features during the differentiation of trNK cells at gd8.5 (Fig. 2i), corroborating a substantial increase in protein synthesis in differentiated trNK cells^55^. Therefore, enhanced ribosome activity likely plays a crucial role in speeding up protein synthesis during trNK cell differentiation, serving as an essential checkpoint in this process. In addition, these findings imply that since our study primarily focuses on gene expression at the mRNA level, it would be worthwhile to conduct a proteomic analysis in the future.

During the differentiation of trNK cells, we have also observed an increase in caspase 3 activity, probably leading to apoptosis of terminally differentiated trNK cells (Fig. 7b, e). This apoptotic process is likely served to prevent the overactivation of uterine trNK. Thus, we tried an inhibition of caspase 3 activity in *Il21r*^-/-^ dams to restore the deficit of terminally differentiated uterine trNK cells. Administering a single dose of caspase 3 inhibitor at gd6.5 to *Il21r*^-/-^ dams results in a partial rescue of the deficit in terminally differentiated trNK cells. In line with it, we have also observed restored spiral artery remodeling and a reduction in fetal resorption (Fig. 7f-j). However, the defect in trNK differentiation at the early stage still remains, suggesting that it is primarily caused by a differentiation defect rather than the caspase 3-mediated apoptotic process. Consistently, the caspase 3 activity in these differentiating trNK cells is not as high as in terminally differentiated trNK cells. From a clinical perspective, it may be worthwhile to further explore whether anti-apoptotic treatment at an appropriate time during gestation could potentially rescue dNK defects and improve reproductive health.

## Methods

### Mice

The animal study protocols used were approved by the Ethics Committee of Peking University Health Science Center. C57BL/6 mice (8–12 weeks old) were purchased from Department of Laboratory Animal Science, Peking University Health Science Center (Beijing100191, China). *Il21r*^-/-^ mice were purchased from the Jackson Laboratory (stock no. 019115). In all matings, female mice were bred with fertility-proven male mice, and the timing of conception was determined by detection of a copulation plug as gd0.5. All animals were bred and maintained in a specific-pathogen-free facility with a 12 h light/12 h dark cycle, an ambient temperature of 20–24 °C, and humidity of 30–70%. All the experimental or control animals were co-housed.

### Tissue digestion and cell preparation

For uterine digestion, the tissues were harvested from virgin or pregnant mice. Then, they were opened longitudinally, cut into small pieces (~5 mm^2^), and incubated for 40 min at 37 °C in RPMI 1640 containing 1 mg/mL collagenase type IV (Sigma-Aldrich), 0.1 mg/mL DNase I (Roche), and 5% FBS. The digested tissues were strained through 40 μm filters, and washed with PBS containing 2% FBS, followed by antibody staining.

### Cell staining, flow cytometry and cell sorting

Digested uterine cells were resuspended in PBS with 2% FBS. Before surface staining, anti-CD16/32 antibody was used to block the Fc receptors. Afterwards, the cells were incubated with antibodies to surface molecules for 30 min at 4 °C, and then washed and resuspended in PBS with 2% FBS. For transcription factor staining, the cells were fixed and permeabilized with Foxp3/Transcription Factor Staining Buffer Set (eBioscience). Then, the cells were stained with antibodies to transcription factors for at least 4 h at 4 °C.

All the antibodies used in this study were listed (Supplementary Table 2). Antibodies specific to mouse CD45.2 (104), CD19 (eBio1D3), CD11b (M1/70), CD11c (N418), CD5 (53-7.3), Gr-1 (RB6-8C5), NKp46 (29A1.4), NK1.1 (PK136), 4-1BB (17B5), EOMES (Dan11mag), Ki-67 (SolA15) were purchased from eBioscience; antibodies specific to mouse CD3 (145-2C11), F4/80 (QA17A29), I-A/I-E (M5/114.15.2), CD49a (HMα1), IL-21R (4A9), CXCR6 (SA051D1), CD16/32 (93) were purchased from BioLegend; antibodies specific to mouse phospho-STAT3 (Tyr705) (4/P-STAT3) were purchased from BD Biosciences; antibody specific to mouse CD55 (076) was purchased from Sino Biological.

Flow cytometric analysis was performed on LSRFortessa (BD Biosciences). Data were analyzed with FlowJo software (BD Biosciences).

Cell sorting was performed on Aria III cytometers (BD Biosciences) at high purity. Uterine trNK cells were sorted by the gating strategy of live CD45^+^ Lin^-^NK1.1^+^NKp46^+^CD49a^+^CXCR6^-^.

### Bulk RNA-sequencing

Uterine trNK from virgin and pregnant C57BL/6 female mice (8–12 weeks old) during decidualization (gd6.5, gd8.5, and gd11.5) were purified by cell sorting and applied to library preparation. In brief, cells were directly sorted to the lysis buffer of SMART-Seq HT Kit (Takara). Reverse transcription and PCR amplification were carried out according to the manufacturer’s instruction. RNA-Seq libraries were prepared using NEB Next Ultra DNA Prep Kit (New England Biolabs). Sample quantity and quality were assessed by Agilent 2100 Bioanalyzer system, and the sequencing was performed on Illumina NovaSeq platform with paired-end 150 base pair reads.

The RNA-seq reads were aligned to the mm10 assembly of mouse genome using HISAT2, and quantified by RSEM. Confirmatively expressed genes (RPKM > 10 in both repeats of any group) were used for the subsequent analysis. Principal component analysis (PCA) and Spearman’s correlation were used for similarity analysis of different trNK groups. Limma pipeline was performed for differential gene expression analysis. GO enrichment analysis was performed through over-representation test using clusterProfiler package. To assess the features of DNA replication, cytolysis, and lymphocyte apoptotic process in trNK, we used a non-parametric and unsupervised software algorithm, Single-sample GSEA (ssGSEA), in GSVA package.

### Single-cell RNA-sequencing

Live CD45^+^NK1.1^+^NKp46^+^ cells in digested uterus from virgin or gd8.5 C57BL/6 female mice (8–12 weeks old) were purified by cell sorting, and labeled by different Sample Tags of BD^TM^ Mouse Immune Single-Cell Multiplexing Kit (BD Biosciences). After washed three times, the samples were mixed. Single-cell capture and cDNA synthesis were conducted using BD Rhapsody^TM^ Single-Cell Analysis System (BD Biosciences), according to the manufacturer’s instruction. The single-cell library was sequenced on Illumina platform.

The single-cell sequencing data were aligned and quantified using the BD Rhapsody WTA Local bioinformatics pipeline (version 1.9.1, BD) against the mouse GRCm39 reference genome. Qualities of the cells were then assessed based on two metrics: (1) The UMI counts per cell should be >200 and <2500; (2) The proportion of mitochondrial genes should be <10%. The UMI counts per cell were normalized by NormalizeData in Seurat package.

For dimension reduction and classification, outliers on a ‘mean variability plot’ were identified by FindVariableFeatures function in Seurat as high variable features. Then, the top 20 high variable feature components were used to calculate PCA matrix with RunPCA function in Seurat. Next, this PCA matrix was applied to build Shared Nearest-neighbor graph using FindNeighbours function. Such Nearest-neighbor graph was used to find cluster by Louvain algorithm with FindClusters function. To identify cluster-specific marker genes, differential expression testing was performed using the FindMarkers function in R package Seurat. Clusters were annotated based on the expression of known marker genes. For dimension reduction, UMAPs were calculated with previous derived PCA matrix using RunUMAP function. For the single-cell analyses in Figs. 5d and 7e, UMAP of trNK in Fig. 2d was utilized as the reference, using FindTransferAnchors and MapQuery functions in Seurat package. Cell trajectory were analyzed by Monocle2 and RNA velocity. For Monocle2, the normalized data from indicated clusters was directly passed to pseudotime inference; for RNA velocity, the velocity vectors were calculated in stochastic model with high variable features derived from pervious step. The activity of gene sets in single cells was calculated using AddModuleScore function in R package Seurat. ScRNA-seq data from human maternal interface was analyzed as the workflow mentioned above.

### ATAC-Sequencing

10,000 sort-purified cells from gd6.5 C57BL/6 dams (8–12 weeks old) were collected and pelleted by centrifugation at 600 g for 10 min. The pellets were resuspended in 50 μL lysis buffer (10 mM Tris-HCl pH 7.4, 10 mM NaCl, 3 mM MgCl_2_, 0.1% NP-40) and kept on ice for 3 min, followed by centrifugating at 600 g for 10 min to collect nuclei. Then, the nuclei pellet was resuspended in transposition reaction buffer. DNA fragmentation and library preparation were performed using TruePrep DNA Library Prep Kit V2 for Illumina (Vazyme) according to the manufacturer’s instruction. Quantity and quality of the libraries were assessed on Agilent 2100 Bioanalyzer system. Library sequencing was performed at paired-end 150 bp on Illumina NovaSeq platform.

ATAC-Seq quality trimming and primer removal were performed with Trimmomatic, using the following parameters: LEADING:15, TRAILING:15, SLIDINGWINDOW:4:15, MINLEN:36. Trimmed reads were aligned to mm10 assembly of mouse genome using Bowtie2. Then, the aligned reads were sorted using samtools, and duplicates were removed using PICARD. Peak-calling for ATAC-Seq was performed with MACS on bam files, with a q-value threshold of 0.01. Consensus peaks from all NK samples were merged to create a raw peak universe. Peak annotation and motif analysis were performed with HOMER. ATAC-Seq tracks were visualized using Integrative Genomics Viewer.

### Cell culture

Uterine trNK from virgin or gd6.5 C57BL/6 female mice (8–12 weeks old) purified by cell sorting were cultured in RPMI 1640 supplied with 10% FBS, 1 mM sodium pyruvate, 10 mM HEPES, 1× penicillin-streptomycin, and 10 ng/mL recombinant murine IL-15 (PeproTech). And, 20 ng/mL recombinant murine IL-21 (BioLegend) and 1 mΜ Stattic (Sigma-Aldrich) were added into the medium as indicated. The cells were cultured for 3 days, followed by flow cytometric analysis or cell sorting.

### STAT3 phosphorylation

Sort-purified uterine trNK from virgin or gd6.5 C57BL/6 female mice (8–12 weeks old) were stimulated by recombinant murine IL-21 (BioLegend) in RPMI 1640 containing 10% fetal bovine serum for 30 min at 37 °C. Then, the cells were fixed by 4% paraformaldehyde for 10 min, and permeabilized with 90% ice-cold methanol for 30 min. After washed twice in PBS, the cells were stained with phospho-STAT3 (Y705) antibody conjugated by Pacific Blue (BD Bioscience) for 4 h at 4 °C. STAT3 phosphorylation was examined by flow cytometry.

### Caspase3 activity detection

Freshly digested uterine cells from gd8.5 C57BL/6 dams (8–12 weeks old) were resuspended in RPMI 1640 containing 10% FBS. To access the activity of caspase 3, TF3-DEVD-FMK (1:250, AAT Bioquest) was added and the cells were cultured for 1 h at 37 °C. Then, the cells were washed with PBS and stained for surface molecules, followed by flow cytometric analysis.

### Caspase3 inhibition and FTY720 treatment in vivo

Caspase3 inhibitor Z-DEVD-FMK (Enzo Life Sciences) and FTY720 were dissolved by 0.9% sterile saline solution. For caspase 3 inhibition, gd6.5 C57BL/6 dams (8–12 weeks old) were intravenously injected with 100 ug Z-DEVD-FMK or vehicle (0.9% sterile saline). For FTY720 treatment, dams were treated daily by 1 mg/kg FTY720 through intraperitoneal injection, from gd4.5 to gd7.5. Mice were sacrificed with a CO_2_ chamber at the indicated time and the uterine tissues were harvested for flow cytometric or histological analysis.

### Intravascular antibody staining

Gd8.5 C57BL/6 dams (8–12 weeks old) were injected with 2 ug FITC conjugated anti-CD45.2 antibody (BioLegend) intravenously. After 3 min, the mice were sacrificed with a CO_2_ chamber to collect uteri for further analysis. Peripheral blood was also collected as a positive control.

### Real-time quantitative PCR

Total RNAs were extracted from uterine tissues by TRIzol reagent (Invitrogen). Reverse transcription and PCR amplification were performed using GoScript™ Reverse Transcription System (Promega), according to the manufacturer’s instruction. The resultant cDNAs were then applied to real-time quantitative PCR with a GoTaq® qPCR and RT-qPCR Systems kit (Promega). Expression of *Hprt* was utilized as an internal control. The following primers are used for the RT-PCR. *Il21*, forward 5’-GCCTCCTGATTAGACTTCGTCAC-3’, reverse 5’-CAGGCAAAAGCTGCATGCTCAC-3’; *Hprt*, forward 5’-CCTAAGATGAGCGCAAGTTGAA-3’, reverse 5’-CCACAGGACTAGAACACCTGCTAA-3’.

### Histology and immunohistochemistry

Embryo implantation sites were isolated from gd8.5 or gd11.5 C57BL/6 dams (8–12 weeks old), and fixed by 4% paraformaldehyde for 24 h. For H&E staining and immunofluorescent analysis, the samples were dehydrated in 20% sucrose, followed by 30% sucrose. Dehydrated samples were embedded in OCT, with subsequent snap frozen. The samples were sectioned (at 10 μm thickness for gd8.5 and 20 μm thickness for gd11.5 embryo implantation sites) and mounted on glass slides. The tissue sections were stained with hematoxylin and eosin. Slide scanning was performed on Nanozoomer digital slide scanner (Hamamatsu). Results were analyzed using NDP.view2 software (Hamamatsu). For immunofluorescent analysis, the sections were incubated in blocking buffer (5% normal donkey serum, 1% BSA, 0.3% Triton X-100 in PBS) for 1 h at room temperature, and then stained with anti-NKp46 (1:25, R&D) and PE conjugated anti-CD55 (1:50, SinoBiological) antibodies in blocking buffer overnight at 4 °C. After washed 3 times in PBS, the tissue sections were stained with Alexa Fluor 488^TM^ conjugated donkey anti-goat secondary antibody (1:1000, Abcam) in blocking buffer for 1 h at room temperature, and counterstained with DAPI for 5 min. The samples were finally mounted and imaged by Nikon N-STORM 4.0 confocal microscope. For immunohistochemical analysis, the fixed samples were dehydrated and embedded in paraffin. 4 μm sections from gd11.5 mice were prepared, and then stained with anti-α-SMA (1:100, BOSTER) overnight at 4 °C. HRP conjugated goat anti-rabbit secondary antibody (1:1000, Abcam) was added, and DAB substrate solution was applied to reveal the smooth muscle actin staining, followed by counterstaining with Hematoxylin. The samples were mounted and imaged by Keyence BZ-X800 microscope.

### Statistical and reproducibility

Differences between two groups were determined by two-sided unpaired Student’s *t*-test using Prism 7 software (GraphPad). *P* < 0.05 was considered as statistically significant. All data were presented as mean ± SEM, *P < 0.05, **P < 0.01, ***P < 0.001. No statistical method was used to predetermine sample size and no data were excluded from the analyses. The mice were randomly divided into different experimental groups or divided by their genotyping and gestational days. Experimental analyses of mice samples were obtained by automated method (flow cytometry, qRT-PCR, et al.) and the investigators were blinded to allocation during experiments and outcome assessment.

### Reporting summary

Further information on research design is available in the Nature Portfolio Reporting Summary linked to this article.

### Supplementary information

Supplementary Information

Description of Additional Supplementary Files

Supplementary Data

Reporting Summary

### Source data

Source Data

## Data Availability

The scRNA-seq, bulk RNA-seq and ATAC-seq data generated in this study are available in the Gene Expression Omnibus (GEO) database (http://www.ncbi.nlm.nih.gov/gds) under the accession number GSE195982. Human scRNA-seq data generated by Vento-Tormo et al. were under accession number E-MTAB-6701. All other data are available in the main text or the Supplementary Information. Source data are provided with this paper.
