# Supplementary material for: IL-21R-STAT3 signalling initiates a differentiation program in uterine tissue-resident NK cells to support pregnancy
Source: Nat Commun. 2023 Nov 4;14:7109. doi: 10.1038/s41467-023-42990-0 (PMC10625623; doi:10.1038/s41467-023-42990-0)
Supplement: Supplementary file 1 — Supplementary Information [file 41467_2023_42990_MOESM1_ESM.pdf]

Supplementary information for

IL-21R-STAT3 signalling initiates a differentiation program in uterine tissue-resident NK cells to support pregnancy

Mengwei Han,[1†] Luni Hu,[1†] Di Wu,[1†], Yime Zhang,[1] Peng Li,[1] Xingyu Zhao,[1] Yanyu Zeng,[1] Guanqun Ren,[1] Zhiyuan Hou,[1] Yanli Pang,[2] Tongbiao Zhao,[3] Chao Zhong[1,4,5*]

[1]Institute of Systems Biomedicine, School of Basic Medical Sciences, Beijing Key Laboratory of Tumor Systems Biology, Peking University Health Science Center, 38 Xueyuan Road, Haidian District, Beijing 100191, China.

[2]Center for Reproductive Medicine, Department of Obstetrics and Gynecology, Peking University Third Hospital, Beijing, China.

[3]National Stem Cell Resource Center, State Key Laboratory of Stem Cell and Reproductive Biology, Institute of Zoology, Institute for Stem Cell and Regeneration, Chinese Academy of Sciences, Beijing, China.

[4]NHC Key Laboratory of Medical Immunology, Peking University, Beijing 100191, China.

[5]Key Laboratory of Molecular Immunology, Chinese Academy of Medical Sciences, Beijing 100191, China.

[†]These authors contributed equally to this work.

*Corresponding author. Email: zhongc@pku.edu.cn (C.Z.)

Supplementary Fig. 1 (related to Fig. 1)

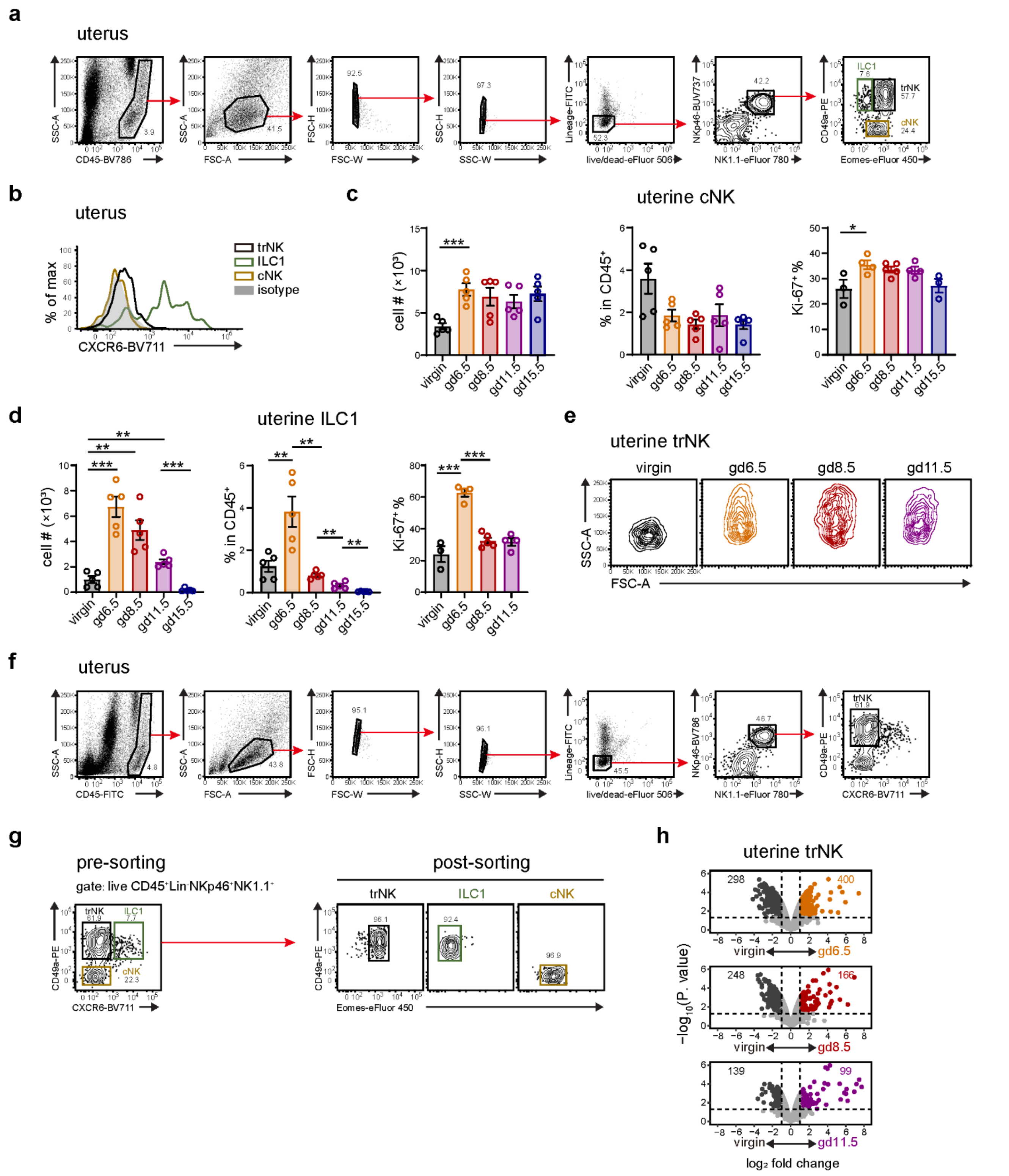

Supplementary Fig. 1. Dynamic changes of uterine trNK, cNK, and ILC1 during decidualization (related to Fig. 1). a Gating strategy for uterine trNK (Eomes$^+$CD49a$^+$), cNK (Eomes$^+$CD49a$^-$), and ILC1 (Eomes$^-$CD49a$^+$) in live CD45$^+$Lin (CD3/CD19/CD5/Gr-1)$^-$ NK1.1$^+$NKp46$^+$ cells. **b** Flow cytometry showing CXCR6 expression on uterine trNK, cNK, and

ILC1. **c** Cell number (n = 5 per group), percentage (in $CD45^+$ immunocytes) (n = 5 per group), and Ki-67 expression of uterine cNK from virgin mice and pregnant mice (from gd6.5 to gd15.5) (n = 3, 4, 5, 4, and 3per group; ***P = 0.0008; *P = 0.0478). **d** Cell number (n = 5 per group; ***P = 0.0001, **P = 0.0014, **P = 0.0031, ***P < 0.0001), percentage (in $CD45^+$ immunocytes) (n = 5 per group; **P = 0.0100, **P = 0.0032, **P = 0.0015, **P = 0.0033), and Ki-67 expression (n = 3, 4, 5, and 4 per group; ***P = 0.0006, ***P < 0.0001) of uterine ILC1 from virgin mice and pregnant mice (from gd6.5 to gd15.5). **e** Flow cytometry showing the changes of uterine trNK in forward and side scatters (FSC-A and SSC-A) during decidualization. **f** Cell sorting gating strategy of uterine trNK ($CD49a^+CXCR6^-$) in live $CD45^+Lin$ $(CD3/CD19/CD5/Gr\text{-}1)^-$ $NK1.1^+NKp46^+$ cells. **g** Flow cytometry showing sorting purity of trNK, ILC1 and cNK. **h** Volcano plots depicting upregulated genes with fold change > 2 and P. value < 0.05 in uterine trNK during decidualization (gd6.5, gd8.5, and gd11.5). Numbers indicate the percentages in each box. Data are shown as the mean ± SEM. P. values are calculated by two-sided unpaired *t*-test, *P < 0.05, **P < 0.01, ***P < 0.001. Data are representative of at least three independent experiments (a-g) or two independent experiments (h). Source data are provided as a Source Data file.

Supplementary Fig. 2 (related to Fig. 2)

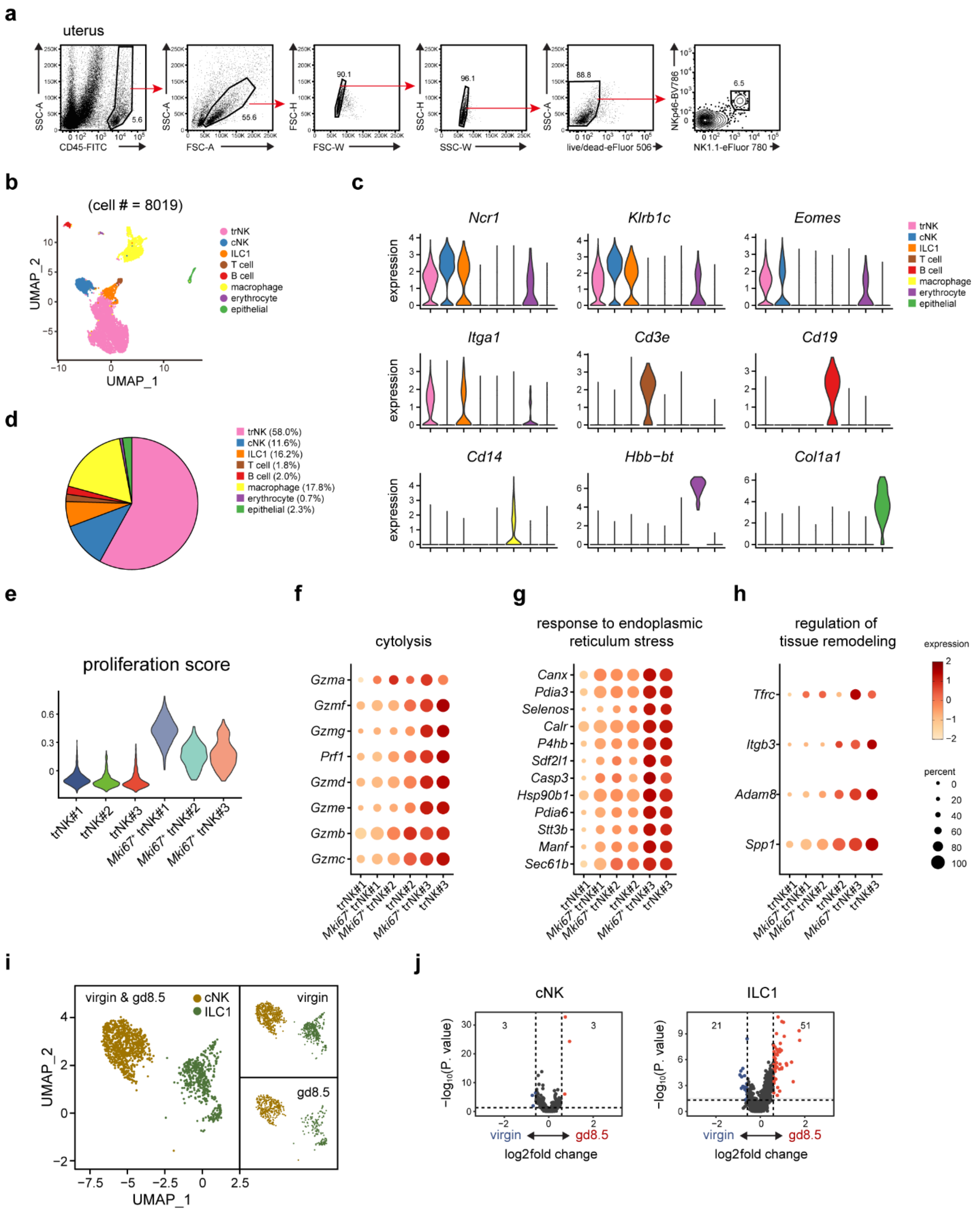

Supplementary Fig. 2. Single-cell transcriptome analysis reveals the changes of uterine trNK, cNK, and ILC1 at gd8.5 (related to Fig. 2). a Gating strategy for NK/ILC1 populations in the uterus. Live CD45^{+}NK1.1^{+}NKp46^{+} NK/ILC1 cells are sorted for single-cell RNA-sequencing. **b** UMAP visualization of CD45^{+}NK1.1^{+}NKp46^{+} cells sorted from uteri of virgin mice

and gd8.5 mice. Colors indicate cell types. **c** Violin plots of selected marker genes for different cell types. **d** Pie chart showing the proportion of each cell type in the uterine sorted cells (b). **e** Single-sample GSEA (ssGSEA) scoring of the proliferation feature in each uterine trNK subgroup. **f** Dot plot of gene ontology for the term of cytolysis in each uterine trNK subgroup. **g** Dot plot of gene ontology for the term of response to endoplasmic reticulum stress in each uterine trNK subgroup. **h** Dot plot of gene ontology for the term of regulation of tissue remodeling in each trNK subgroup. **i** UMAP visualization of uterine cNK and ILC1 in virgin mice and gd8.5 mice. **j** Volcano plots showing differentially expressed genes in uterine cNK and ILC1 from virgin mice and gd8.5 mice. Data are representative of at least three independent experiments (a) or two independent experiments (b-j).

Supplementary Fig. 3 (related to Fig. 3)

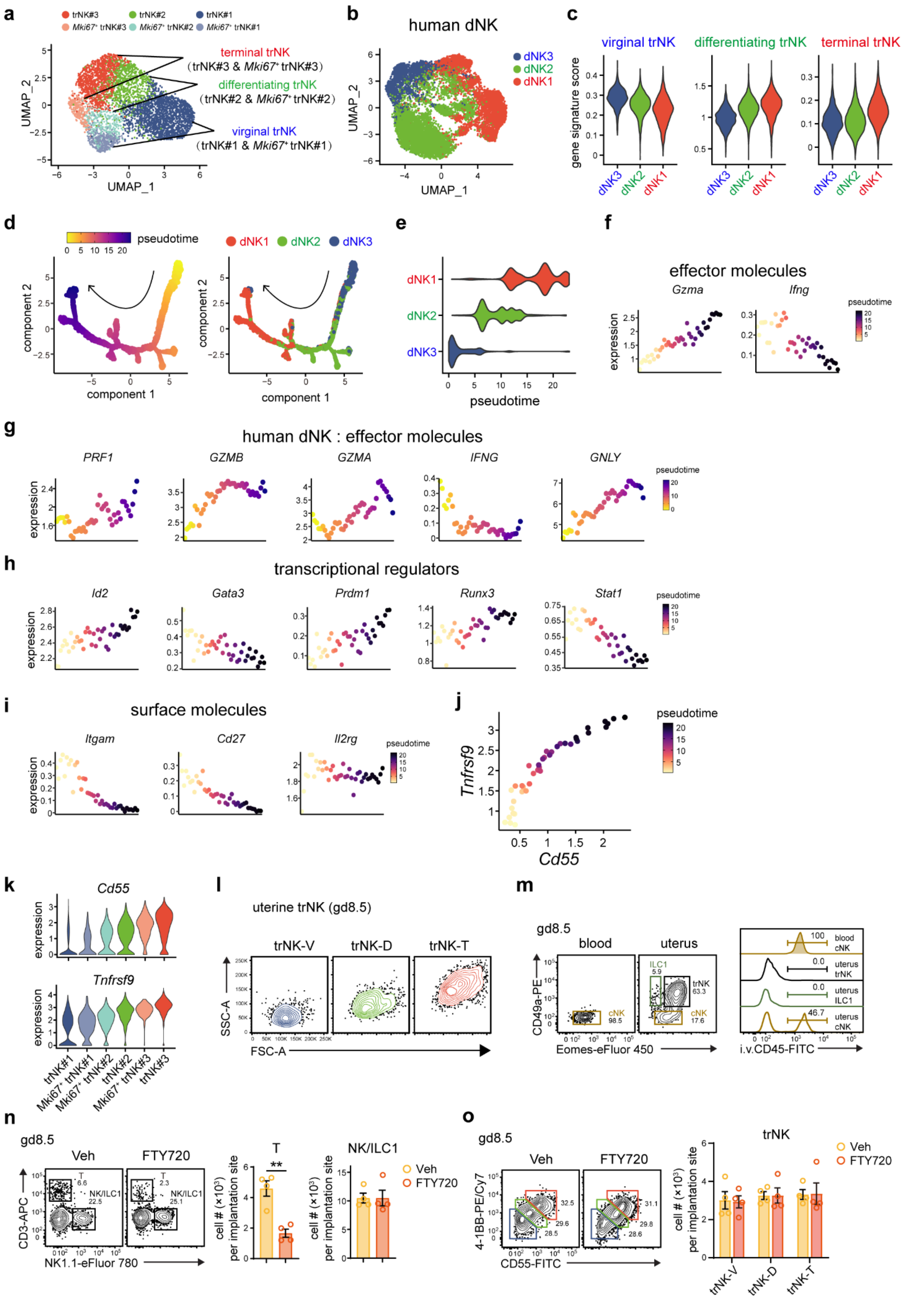

Supplementary Fig. 3. 4-1BB and CD55 denote the differentiation trajectory of uterine trNK (related to Fig. 3). a Nomenclature of uterine trNK subgroups at different differentiation stages. (b-e, g) Re-analysis of public scRNA-seq data of human decidual NK (dNK) (#E-MTAB-6701). **b** UMAP of human dNK subgroups. **c** Scoring of each human dNK subgroup by the gene signatures of murine uterine trNK subgroups. **d** Pseudotime of human dNK subgroups (left), and their distribution along the trajectory (right). **e** Separated distribution human dNK subgroups along the pseudotime trajectory. **f** Dynamic expression of the indicated effector genes in gd8.5 murine uterine trNK, respected to the pseudotime coordinates. **g** Dynamic expression of the indicated effector genes in human dNK, respected to the pseudotime coordinates. **h** Dynamic expression of the indicated transcription regulators in gd8.5 murine uterine trNK, respected to the pseudotime coordinates. **i** Dynamic expression of the indicated surface molecules in gd8.5 murine uterine trNK, respected to the pseudotime coordinates. **j** Correlation of the expression of *Tnfrsf9* and *Cd55* in gd8.5 murine uterine trNK, respected to the pseudotime coordinates. **k** Violin plots showing expression of *Tnfrsf9* and *Cd55* in each gd8.5 uterine trNK subgroup. **l** Flow cytometry showing forward and side scatters of gd8.5 uterine trNK subgroups. **m** Flow cytometry showing cNK from blood, and cNK, trNK and ILC1 from gd8.5 uterus. Intravascular staining by intravenously injection of FITC conjugated anti-CD45 antibody has lasted for 3 minutes, followed by mice sacrifice. **n** Flow cytometric and statistical analysis of uterine T cells ($CD3^{+}NK1.1^{-}$) and NK/ILC1 ($CD3^{-}NK1.1^{+}$) in gd8.5 mice with FTY720 or vehicle (saline) administrated 4 days before (n = 4 per group; **P = 0.0021). **o** Flow cytometric and statistical analysis of uterine trNK subgroups in gd8.5 mice with FTY720 or vehicle administrated 4 days before (n = 4 per group). Numbers indicate the percentages in each box. Data are shown as the mean ± SEM. P. values are calculated by two-sided unpaired *t*-test, **P < 0.01. Data are representative of two independent experiments (a, f and h-k) or at least three independent experiments (l-o). Source data are provided as a Source Data file.

Supplementary Fig. 4 (related to Fig. 4)

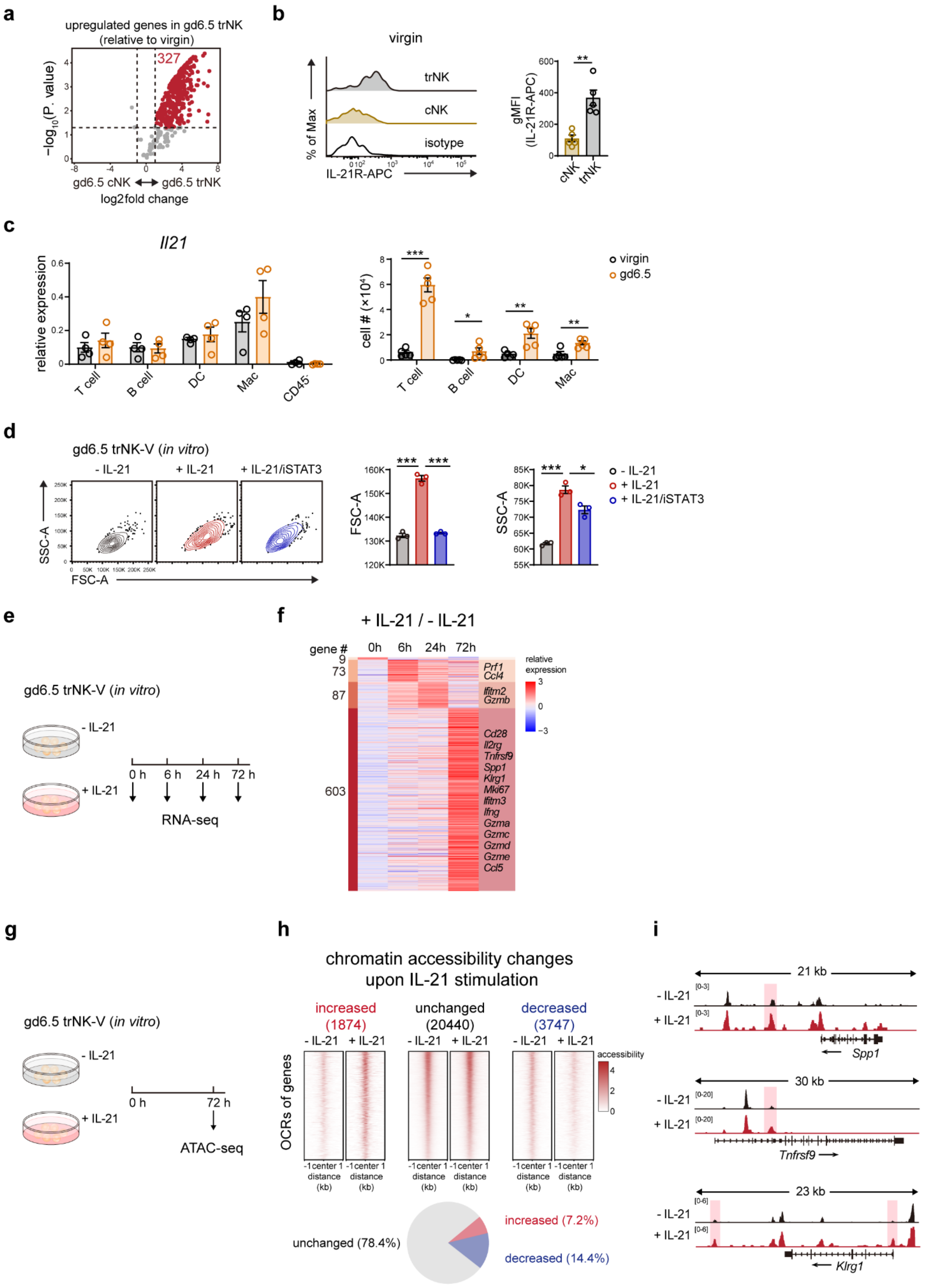

Supplementary Fig. 4. IL-21R-STAT3 axis initiates uterine trNK differentiation (related to Fig. 4). a Volcano plots depicting differentially expressed genes (fold change > 2, P value < 0.05) between gd6.5 uterine trNK and cNK. The genes are from the upregulated genes in uterine trNK of gd6.5 dams compared to virgin mice (related to Supplementary Fig. 1h). **b** Flow cytometric and statistical analysis of IL-21R expression on uterine trNK and cNK from virgin mice (n = 5 per group; **P = 0.0013). **c** Expression of *Il21* (relative to *Hprt*) (n = 4 per group) and cell number (n = 5 per group; ***P < 0.0001, *P = 0.0396, **P = 0.0043, **P = 0.0062) of the indicated cell types in uteri of virgin and gd6.5 mice. **d** Flow cytometry and statistical analysis showing the forward and side scatters of gd6.5 trNK-V subgroup stimulated in the indicated conditions for 72 hours (n = 3 per group; ***P < 0.0001, ***P < 0.0001; ***P = 0.0002, *P = 0.0204). iSTAT3, STAT3 inhibitor. **e** Schematic diagram showing sorted-purified gd6.5 trNK-V subgroup cultured and harvested for RNA-seq under the indicated conditions. **f** Heatmap showing the relative gene expression differences between IL-21 treated and untreated trNK at the indicated time-points. **g** Schematic diagram showing sorted-purified gd6.5 trNK-V subgroup cultured in presence or absence of recombinant IL-21, followed by ATAC-seq analysis at 72h. **h** Heatmaps of ATAC-seq analysis depicting the open chromatin regions (OCRs) in gd6.5 trNK-V subgroup with or without IL-21 stimulation for 72 hours. OCRs demonstrating increased, unchanged or decreased chromatin accessibility upon IL-21 stimulation are grouped and their proportions are calculated. **i** Representative ATAC-seq tracks at the *Spp1*, *Tnfrsf9* and *Klrg1* gene loci, with the peak differences highlighted in pink. Numbers indicate the percentages in each box. Data are shown as the mean ± SEM. P. values are calculated by two-sided unpaired *t*-test, *P < 0.05, **P < 0.01, ***P < 0.001. Data are representative of two independent experiments (a, e-i) or at least three independent experiments (b-d). Source data are provided as a Source Data file.

Supplementary Fig. 5 (related to Fig. 5)

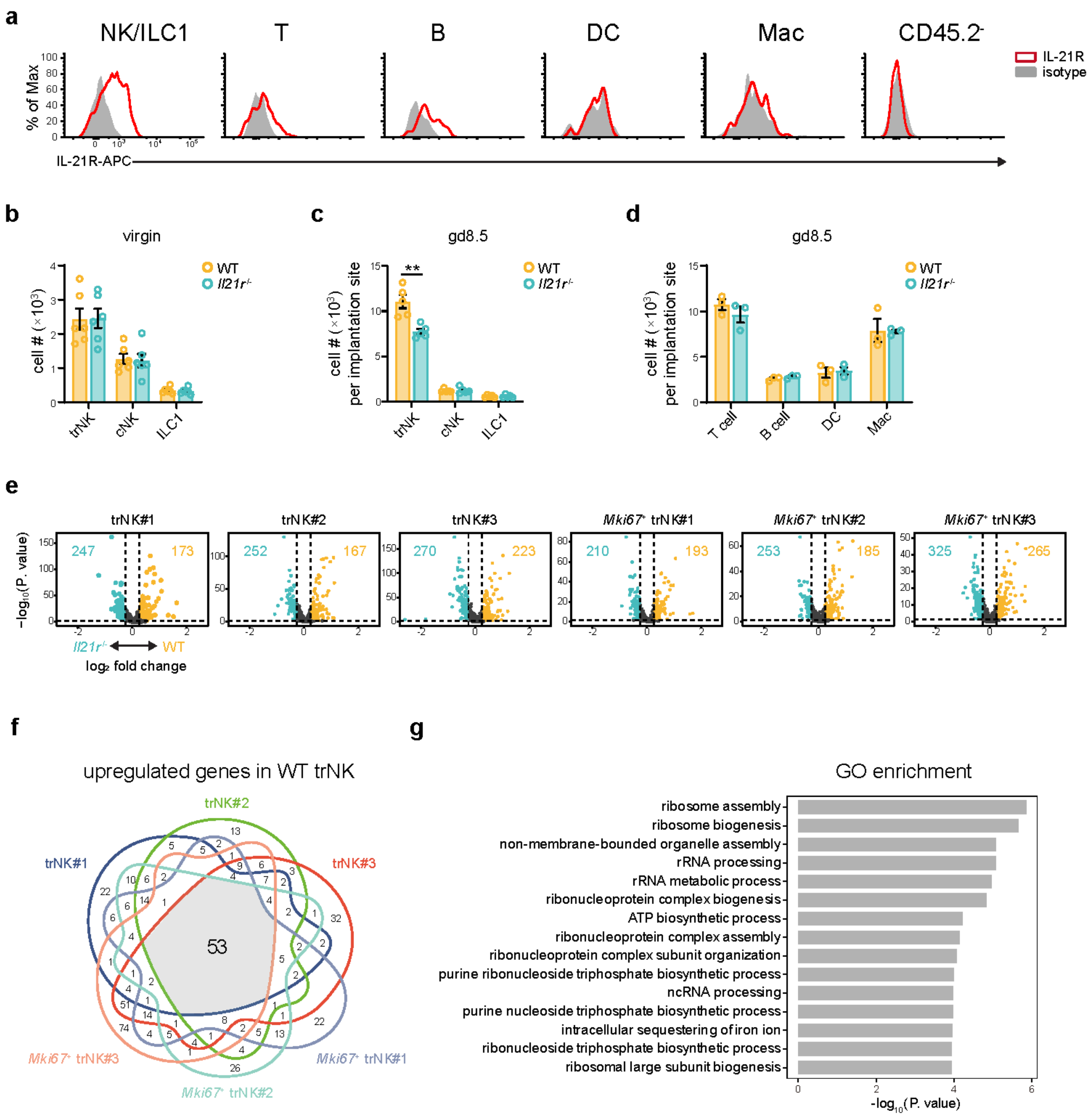

Supplementary Fig. 5. Uterine trNK differentiation is affected by *Il21r* deficiency (related to Fig. 5). a Flow cytometry showing IL-21R expression on different cell types in the uterus, including CD45⁻ cells, NK/ILC1, T cells, B cells, dendritic cells (DC) and macrophages (Mac). **b** Cell number comparisons of uterine trNK, cNK, and ILC1 in wild-type (WT) and *Il21r* $^{-/-}$ mice before pregnancy (n = 6 per group). **c** Cell number comparisons of uterine trNK, cNK, and ILC1 in WT and *Il21r*$^{-/-}$ mice at gd8.5 (n = 5 per group; **P = 0.0031). **d** Cell number comparisons of the indicated immunocytes in uteri of WT and *Il21r*$^{-/-}$ mice at gd8.5 (n = 3 per group). **e** Volcano plots depicting differentially expressed genes ($\log_2$ fold change > 0.25 and P. value < 0.05) in each uterine trNK subgroup between WT and *Il21r*$^{-/-}$ mice at gd8.5. **f** Venn diagram showing concordantly upregulated genes in uterine trNK of WT versus *Il21r*$^{-/-}$ mice. **g** GO enrichment of the concordantly upregulated genes in uterine trNK of WT versus *Il21r*$^{-/-}$

mice. Numbers indicate the percentages in each box. Data are shown as the mean ± SEM. P. values are calculated by two-sided unpaired *t*-test, **P < 0.01. Data are representative of at least three independent experiments (a-d) or two independent experiments (e-g). Source data are provided as a Source Data file.

Supplementary Fig. 6 (related to Fig. 6)

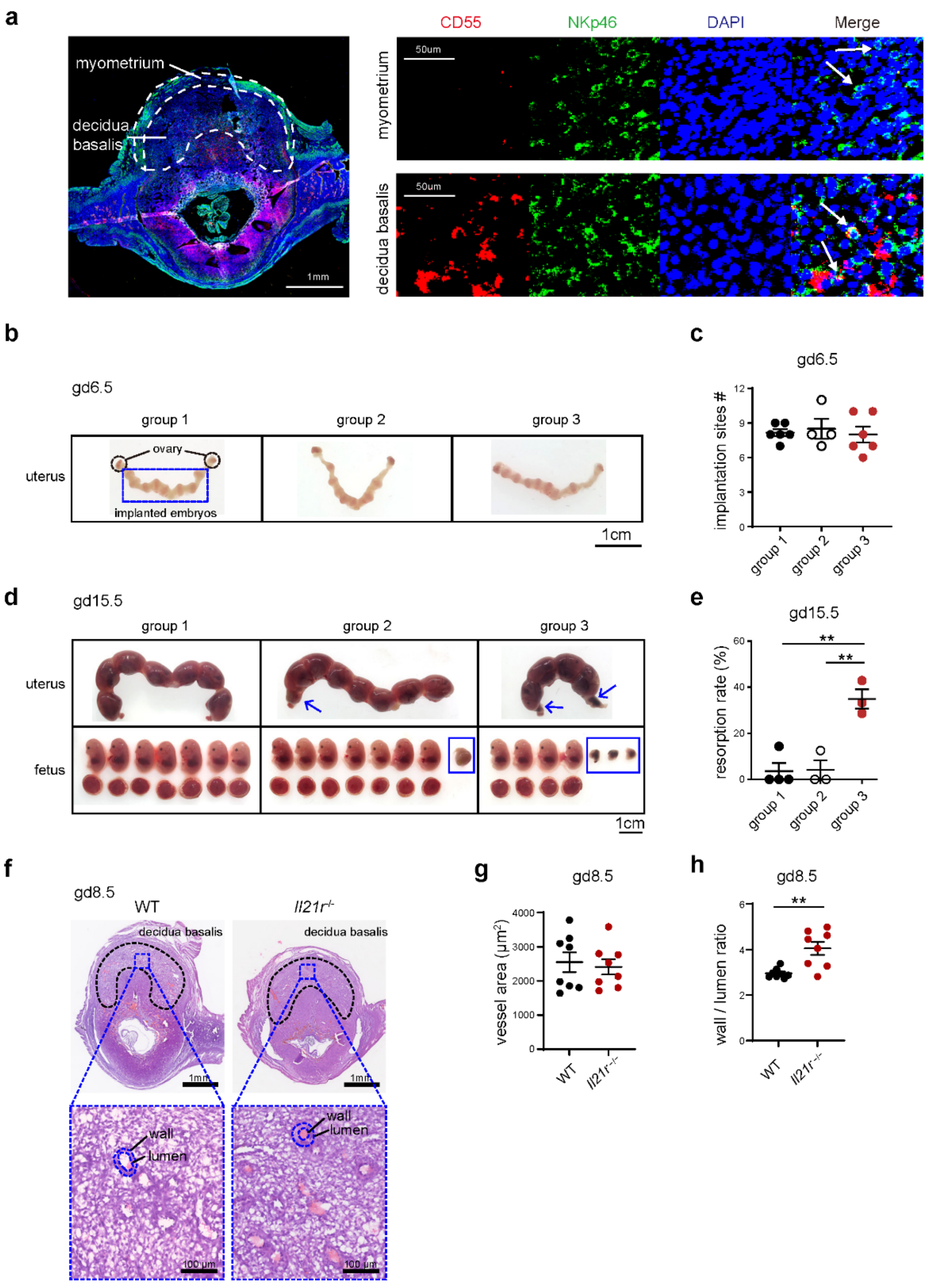

Supplementary Fig. 6. *Il21r* deficiency causes increased incidence of miscarriage and defective spiral artery remodeling (related to Fig. 6). a Confocal microscopy showing positioning of differentiated uterine trNK in decidua basalis. The image in left represents a whole implantation site of gd8.5 mice. Positioning of the CD55 expressing or non-expressing NKp46$^+$ NK cells (white arrows) in myometrium and decidua basalis is further analyzed in right at high magnification. **b** Embryo Implantation in gd6.5 dams of groups 1, 2 and 3 (related to Fig. 6a). Ovaries (dashed black lines) locate at two ends of the uterus, and implantation sites

(dashed blue lines) distribute between them. **c** Number of implantation sites in gd6.5 dams of groups 1, 2 and 3 (n = 6, 4, and 6 per group). **d** Implanted embryos in uteri of gd15.5 mice from groups 1, 2, and 3. Resorbed fetuses (Blue arrows and bule rectangles) are distinguished in groups 2 and 3. Scale bar = 1 cm. **e** Resorption rate in gd15.5 dams of groups 1, 2, and 3 (n = 4, 3, and 3 per group; **P = 0.0023, **P = 0.0065), calculated by (the number of resorptions) / (the number of resorptions and successful implantations). **f** H&E staining showing whole implantation sites in WT and *Il21r*$^{-/-}$ dams at gd8.5. Spiral artery remodeling (dashed blue circles) in decidua basalis (dashed black lines) is assessed by the wall and lumen areas. **g** Vessel wall areas of spiral arteries in gd8.5 dams (n = 8 per group). Each data point represents mean of five measurements in an implantation site. **h** Ratios between wall area and the corresponding lumen area of spiral artery vessels in gd8.5 dams (n = 8 per group; **P = 0.0023). Each data point represents mean of five measurements in an implantation site. Data are shown as the mean ± SEM. P. values are calculated by two-sided unpaired *t*-test, **P < 0.01. Data are representative of at least three independent experiments (a-h). Source data are provided as a Source Data file.

Supplementary Fig. 7 (related to Fig. 7)

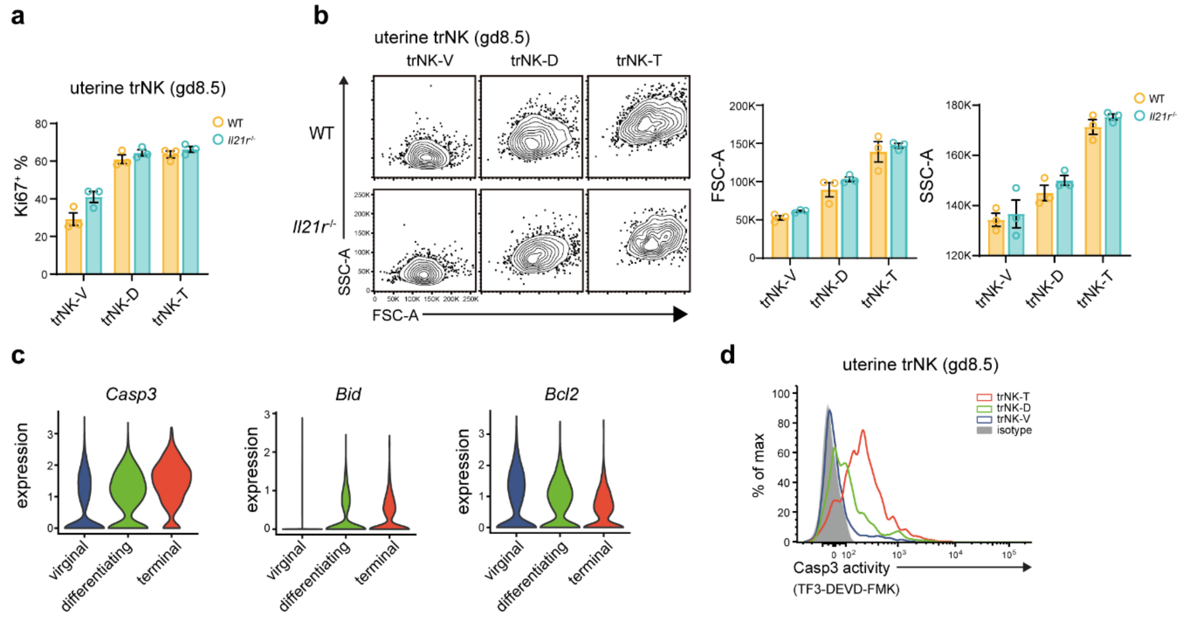

Supplementary Fig. 7. An apoptotic process is initiated along with uterine trNK differentiation (related to Fig. 7). a Percentage of Ki-67$^+$ in the trNK-V, trNK-D and trNK-T from WT and *Il21r*$^{-/-}$ mice at gd8.5 (n = 3 per group). **b** Flow cytometry and statistical analysis showing the changes of forward and side scatters (FSC-A and SSC-A) in the uterine trNK-V, trNK-D and trNK-T from WT and *Il21r*$^{-/-}$ mice at gd8.5 (n = 3 per group). **c** Violin plots showing expression of pro-apoptotic genes, *Casp3* and *Bid*, and anti-apoptotic gene *Bcl2* for virginal, differentiating, and terminal trNK subgroups. **d** Flow cytometric histogram showing caspase 3 activities in the uterine trNK-V, trNK-D and trNK-T at gd8.5. Numbers indicate the percentages in each box. Data are shown as the mean ± SEM. Numbers indicate the percentages in each box. Data are representative of at least three independent experiments (a, b, and d) or two independent experiments (c). Source data are provided as a Source Data file.

Supplementary Table 1. Statistics of apoptosis-related genes at different trNK differentiation stages (related to Fig 7a). Results are generated by the FindMarkers function from Seurat package using Wilcoxon Rank Sum test. The table contains columns for average gene expression in WT (WT_mean_expr) and KO (*Il21r*$^{-/-}$_mean_expr), Wilcox test p value (wilcox_p_val), and average log2 fold change (avg_log2FC).

Gene	Stage	WT_mean_expr	*Il21r*$^{-/-}$_mean_expr	wilcox_p_val	avg_log2FC
Casp3	virginal trNK	0.737191353	0.678478603	0.152292509	0.214503281
	differentiating trNK	1.037249731	1.001407495	0.107440051	0.159147747
	terminal trNK	1.430406438	1.150014511	3.74877E-24	0.49389674
Bid	virginal trNK	0.24594467	0.295542069	8.96151E-07	-0.024399409
	differentiating trNK	0.330172059	0.386557983	9.42488E-05	-0.028959901
	terminal trNK	0.341635174	0.378226118	0.002527661	-0.019977544
Bcl2	virginal trNK	0.869587185	1.035424412	6.017E-08	-0.145785579
	differentiating trNK	0.79679648	0.89184358	0.002619439	-0.036964254
	terminal trNK	0.570675784	0.612825928	0.034258056	-0.016676566

Supplementary Table 2. Antibodies used in this study

Antibody	Dilution	Catalogue number
anti-mouse NK1.1 APC-eFluor™ 780	1:300	47-5941-82
anti-mouse CD137 (4-1BB) PE/Cyanine7	1:400	25-1371-82
anti-mouse EOMES eFluor™ 450	1:400	48-4875-82
anti-mouse Ki-67 Alexa Fluor™ 700	1:400	56-5698-82
nti-mouse CD11b eFluor™ 450	1:400	48-0112-82
anti-mouse CD16/32	1:100	101319
anti-mouse CD3e FITC	1:1000	100306
anti-mouse CD19 FITC	1:1000	115506
anti-mouse CD5 FITC	1:1000	100606
anti-mouse Ly-6G/Ly-6C FITC	1:1000	108406
anti-mouse CD11c FITC	1:1000	117323
anti-mouse CD45.2 Brilliant Violet 785™	1:400	109839
anti-mouse CD45.2 FITC	1:400	109806
anti-mouse CD49a PE	1:400	142604
anti-mouse IL-21R APC	1:300	131910
anti-mouse CXCR6 Brilliant Violet 711™	1:400	151111
anti-mouse I-A/I-E Brilliant Violet 650™	1:400	107641
anti-mouse F4/80 PE/Cyanine7	1:400	157308
anti-mouse CD3e PE/Cyanine7	1:400	100320
anti-mouse NKp46 Biotin	1:300	137616
anti-mouse CD19 Biotin	1:400	115504
Brilliant Violet 785™ Streptavidin	1:400	405249
anti-mouse p-STAT3(pY705) Pacific Blue™	1:100	560312
BUV737 Streptavidin	1:400	564293
anti-mouse CD55 FITC	1:100	50468-R076-F
Fixable Viability Dye eFluor™ 506	1:1000	65-0866-14
anti-mouse CD55 PE	1:50	50468-R076-P
anti-mouse NKp46	1:25	AF2225-SP
Donkey anti-goat IgG H&L-Alexa Fluor 488	1:1000	ab150129
anti-mouse α-SMA	1:100	BM0002
Goat anti-rabbit IgG H&L-HRP	1:1000	ab6721
