# Supplementary material for: IL-21R-STAT3 signalling initiates a differentiation program in uterine tissue-resident NK cells to support pregnancy
Source: Nat Commun. 2023 Nov 4;14:7109. doi: 10.1038/s41467-023-42990-0 (PMC10625623; doi:10.1038/s41467-023-42990-0)
Supplement: Supplementary file 2 — Description of Additional Supplementary Files [file 41467_2023_42990_MOESM2_ESM.pdf]

Description of Additional Supplementary Files

File Name: Supplementary Data
Description: **Differential gene expression analysis of overall trNK cells and each individual trNK clusters between WT and *Il21r-/-* mice.** Results are generated by the FindMarkers function from Seurat package using Wilcoxon Rank Sum test. The table contains columns for p value (p_val), average log2 fold change (avg_log2FC), expression percentage in WT (pct.1) and *Il21r-/-* (pct.2), and gene classification (class). Each table represents the indicated comparison. Related to Fig. 5h and S5e.
