# Supplementary material for: IL-21R-STAT3 signalling initiates a differentiation program in uterine tissue-resident NK cells to support pregnancy
Source: Nat Commun. 2023 Nov 4;14:7109. doi: 10.1038/s41467-023-42990-0 (PMC10625623; doi:10.1038/s41467-023-42990-0)
Supplement: Supplementary file 4 — Reporting Summary [file 41467_2023_42990_MOESM4_ESM.pdf]

nature portfolio

Corresponding author(s): Chao Zhong

Last updated by author(s): Oct 12, 2023

Reporting Summary

Nature Portfolio wishes to improve the reproducibility of the work that we publish. This form provides structure for consistency and transparency in reporting. For further information on Nature Portfolio policies, see our Editorial Policies and the Editorial Policy Checklist.

Statistics

For all statistical analyses, confirm that the following items are present in the figure legend, table legend, main text, or Methods section.

n/a	Confirmed	
☐	☒	The exact sample size (*n*) for each experimental group/condition, given as a discrete number and unit of measurement
☐	☒	A statement on whether measurements were taken from distinct samples or whether the same sample was measured repeatedly
☐	☒	The statistical test(s) used AND whether they are one- or two-sided<br>*Only common tests should be described solely by name; describe more complex techniques in the Methods section.*
☐	☒	A description of all covariates tested
☐	☒	A description of any assumptions or corrections, such as tests of normality and adjustment for multiple comparisons
☐	☒	A full description of the statistical parameters including central tendency (e.g. means) or other basic estimates (e.g. regression coefficient) AND variation (e.g. standard deviation) or associated estimates of uncertainty (e.g. confidence intervals)
☐	☒	For null hypothesis testing, the test statistic (e.g. *F*, *t*, *r*) with confidence intervals, effect sizes, degrees of freedom and *P* value noted<br>*Give P values as exact values whenever suitable.*
☒	☐	For Bayesian analysis, information on the choice of priors and Markov chain Monte Carlo settings
☒	☐	For hierarchical and complex designs, identification of the appropriate level for tests and full reporting of outcomes
☒	☐	Estimates of effect sizes (e.g. Cohen's *d*, Pearson's *r*), indicating how they were calculated

Our web collection on statistics for biologists contains articles on many of the points above.

Software and code

Policy information about availability of computer code

Data collection	The RNA-seq and ATAC-seq data: Illumina NovaSeq platform<br>The Flow cytometric data: LSRFortessa and FACSAria™ III (BD Biosciences)<br>Immunofluorescent images: Nikon N-STORM 4.0 confocal microscope<br>Histopathological Images: Nanozoomer digital slide scanner (Hamamatsu)
Data analysis	Statistical analysis: Graphpad Prism version 9.0.2<br>Flow cytometric analysis: Flowjo software 10.5.3<br>RNA-seq analysis: limma v3.46.0, clusterProfiler v3.18.0, GSVA v1.38.0, Seurat v4.0.0, R software v4.0.3<br>Immunofluorescence analysis: NIS-Elements Viewer 5.21<br>Histopathological image analysis: NDP.view2 software v2.7.52

For manuscripts utilizing custom algorithms or software that are central to the research but not yet described in published literature, software must be made available to editors and reviewers. We strongly encourage code deposition in a community repository (e.g. GitHub). See the Nature Portfolio guidelines for submitting code & software for further information.

Data

Policy information about availability of data

All manuscripts must include a data availability statement. This statement should provide the following information, where applicable:

- Accession codes, unique identifiers, or web links for publicly available datasets
- A description of any restrictions on data availability
- For clinical datasets or third party data, please ensure that the statement adheres to our policy

The scRNA-seq, bulk RNA-seq and ATAC-seq data generated in this study are available in the Gene Expression Omnibus (GEO) database (http://www.ncbi.nlm.nih.gov/gds) under the accession number GSE195982 [https://www.ncbi.nlm.nih.gov/geo/query/acc.cgi?acc=GSE195982]. Human scRNA-seq data generated by Vento-Tormo et al were under accession number E-MTAB-6701. All other data are available in the main text or the Supplementary Information. Source data are provided with this paper.

Research involving human participants, their data, or biological material

Policy information about studies with human participants or human data. See also policy information about sex, gender (identity/presentation), and sexual orientation and race, ethnicity and racism.

Reporting on sex and gender	n/a
Reporting on race, ethnicity, or other socially relevant groupings	n/a
Population characteristics	n/a
Recruitment	n/a
Ethics oversight	n/a

Note that full information on the approval of the study protocol must also be provided in the manuscript.

Field-specific reporting

Please select the one below that is the best fit for your research. If you are not sure, read the appropriate sections before making your selection.

☒ Life sciences ☐ Behavioural & social sciences ☐ Ecological, evolutionary & environmental sciences

For a reference copy of the document with all sections, see nature.com/documents/nr-reporting-summary-flat.pdf

Life sciences study design

All studies must disclose on these points even when the disclosure is negative.

Sample size	Sample sizes were chosen based on preliminary data demonstrating statistically significant differences for each specific assay.
Data exclusions	No data were excluded from the analysis.
Replication	The experimental findings were reliably reproduced. The replication numbers were described in the corresponding figure legends.
Randomization	Age-matched female mice were assigned randomly to experimental and control groups. Other experimental allocation is random.
Blinding	Experimental analyses of mice samples were obtained by automated method (flow cytometry, qRT-PCR, et al.) and the investigators were blinded to allocation during experiments and outcome assessment.

Reporting for specific materials, systems and methods

We require information from authors about some types of materials, experimental systems and methods used in many studies. Here, indicate whether each material, system or method listed is relevant to your study. If you are not sure if a list item applies to your research, read the appropriate section before selecting a response.

Materials & experimental systems

n/a	Involved in the study
☐	☒ Antibodies
☒	☐ Eukaryotic cell lines
☒	☐ Palaeontology and archaeology
☐	☒ Animals and other organisms
☒	☐ Clinical data
☒	☐ Dual use research of concern
☒	☐ Plants

Methods

n/a	Involved in the study
☒	☐ ChIP-seq
☐	☒ Flow cytometry
☒	☐ MRI-based neuroimaging

Antibodies

Antibodies used

Flow cytometry:
anti-mouse NK1.1 APC-eFluor™ 780, Thermo Fisher Scientific, Cat No 47-5941-82, clone PK136
anti-mouse CD137 (4-1BB) PE/Cyanine7, Thermo Fisher Scientific, Cat No 25-1371-82, clone 17B5
anti-mouse EOMES eFluor™ 450, Thermo Fisher Scientific, Cat No 48-4875-82, clone Dan11mag
anti-mouse Ki-67 Alexa Fluor™ 700, Thermo Fisher Scientific, Cat No 56-5698-82, clone SolA15
anti-mouse CD11b eFluor™ 450, Thermo Fisher Scientific, Cat No 48-0112-82, clone M1/70
anti-mouse CD16/32, Biolegend, Cat No 101319, clone 93
anti-mouse CD3e FITC, Biolegend, Cat No 100306, clone 145-2C-11
anti-mouse CD19 FITC, Biolegend, Cat No 115506, clone 6D5
anti-mouse CD5 FITC, Biolegend, Cat No 100606, clone 53-7.3
anti-mouse Ly-6G/Ly-6C FITC, Biolegend, Cat No 108406, clone RB6-8C5
anti-mouse CD11c FITC, Biolegend, Cat No 117323, clone N418
anti-mouse CD45.2 Brilliant Violet 785™, Biolegend, Cat No 109839, clone 104
anti-mouse CD45.2 FITC, BioLegend, Cat No 109806, clone 104
anti-mouse CD49a PE, BioLegend, Cat No 142604, clone HMα1
anti-mouse IL-21R APC, BioLegend, Cat No 131910, clone 4A9
anti-mouse CXCR6 Brilliant Violet 711™, BioLegend, Cat No 151111, clone SA0511D1
anti-mouse I-A/I-E Brilliant Violet 650™, BioLegend, Cat No 107641, clone M5/114.152
anti-mouse F4/80 PE/Cyanine7, BioLegend, Cat No 157308, clone QA17A29
anti-mouse CD3e PE/Cyanine7, BioLegend, Cat No 100320, clone 145-2C11
anti-mouse NKp46 Biotin, Biolegend, Cat No 137616, clone 29A1.4
anti-mouse CD19 Biotin, BioLegend, Cat No 115504, clone 6D5
Brilliant Violet 785™ Streptavidin, BioLegend, Cat No 405249
anti-mouse p-STAT3(pY705) Pacific Blue™, BD Biosciences, Cat No 560312, clone 4/P-STAT3
BUV737 Streptavidin, BD Biosciences, Cat No 564293
anti-mouse CD55 FITC, Sino Biological, Cat No 50468-R076-F, clone 076
Fixable Viability Dye eFluor™ 506, Thermo Fisher Scientific, Cat No 65-0866-14
Immunohistochemistry:
anti-mouse CD55 PE, Sino Biological, Cat No 50468-R076-P, clone 076
anti-mouse NKp46, R&D, Cat No AF2225-SP, Polyclone
Donkey anti-goat IgG H&L-Alexa Fluor 488, Abcam, Cat No ab150129
anti-mouse α-SMA, BOSTER, Cat No BM0002, clone 1A4
Goat anti-rabbit IgG H&L-HRP, Abcam, Cat No ab6721

Validation

All antibodies are commercially available and have validated by the manufacturers. Specific validation information can be found on their respective website as below:
anti-mouse NK1.1 APC-eFluor™ 780: https://www.thermofisher.cn/cn/zh/antibody/product/NK1-1-Antibody-clone-PK136-Monoclonal/47-5941-82
anti-mouse CD137 (4-1BB) PE/Cyanine7: https://www.thermofisher.cn/cn/zh/antibody/product/CD137-4-1BB-Antibody-clone-17B5-Monoclonal/25-1371-82
anti-mouse EOMES eFluor™ 450: https://www.thermofisher.cn/cn/zh/antibody/product/EOMES-Antibody-clone-Dan11mag-Monoclonal/48-4875-82
anti-mouse Ki-67 Alexa Fluor™ 700: https://www.thermofisher.cn/cn/zh/antibody/product/Ki-67-Antibody-clone-SolA15-Monoclonal/56-5698-82
anti-mouse CD11b eFluor™ 450: https://www.thermofisher.cn/cn/zh/antibody/product/CD11b-Antibody-clone-M1-70-Monoclonal/48-0112-82
anti-mouse CD16/32: https://www.biolegend.com/en-us/products/trustain-fcx-anti-mouse-cd16-32-antibody-5683
anti-mouse CD3e FITC: https://www.biolegend.com/en-us/products/fitc-anti-mouse-cd3epsilon-antibody-23
anti-mouse CD19 FITC: https://www.biolegend.com/en-us/products/fitc-anti-mouse-cd19-antibody-1528
anti-mouse CD5 FITC: https://www.biolegend.com/en-us/products/fitc-anti-mouse-cd5-antibody-159
anti-mouse Ly-6G/Ly-6C FITC:https://www.biolegend.com/en-us/products/fitc-anti-mouse-ly-6g-ly-6c-gr-1-antibody-458
anti-mouse CD11c FITC: https://www.biolegend.com/en-us/products/fitc-anti-mouse-cd11c-antibody-1815
anti-mouse CD45.2 Brilliant Violet 785™: https://www.biolegend.com/en-us/products/brilliant-violet-785-anti-mouse-cd45-2-antibody-8924
anti-mouse CD45.2 FITC: https://www.biolegend.com/en-us/products/fitc-anti-mouse-cd45-2-antibody-6
anti-mouse CD49a PE: https://www.biolegend.com/en-us/products/pe-anti-mouse-cd49a-antibody-7535
anti-mouse IL-21R APC: https://www.biolegend.com/en-us/products/apc-anti-mouse-il-21r-antibody-5521
anti-mouse CXCR6 Brilliant Violet 711™: https://www.biolegend.com/en-us/products/brilliant-violet-711-anti-mouse-cd186-cxcr6-

antibody-15094
anti-mouse I-A/I-E Brilliant Violet 650™: https://www.biolegend.com/en-us/products/brilliant-violet-650-anti-mouse-i-a-i-e-antibody-12085
anti-mouse F4/80 PE/Cyanine7: https://www.biolegend.com/en-us/products/pecyanine7-anti-mouse-f480-recombinant-antibody-18757
anti-mouse CD3e PE/Cyanine7: https://www.biolegend.com/en-us/products/pe-cyanine7-anti-mouse-cd3epsilon-antibody-1899
anti-mouse NKp46 Biotin: https://www.biolegend.com/en-us/products/biotin-anti-mouse-cd335-nkp46-antibody-7898
anti-mouse CD19 Biotin: https://www.biolegend.com/en-us/products/biotin-anti-mouse-cd19-antibody-1527
Brilliant Violet 785™ Streptavidin: https://www.biolegend.com/en-us/products/brilliant-violet-785-streptavidin-10344
anti-mouse p-STAT3(pY705) Pacific Blue™: https://www.bdbiosciences.com/zh-cn/products/reagents/flow-cytometry-reagents/research-reagents/single-color-antibodies-ruo/pacific-blue-mouse-anti-stat3-py705.560312
BUV737 Strptavidin: https://www.bdbiosciences.com/zh-cn/products/reagents/flow-cytometry-reagents/research-reagents/single-color-antibodies-ruo/buv737-streptavidin.612775
anti-mouse CD55 FITC: https://www.sinobiological.com/antibodies/mouse-cd55-daf-50468-r076-p
Fixable Viability Dye eFluor™ 506: https://www.thermofisher.cn/order/catalog/product/65-0866-14?SID=srch-srp-65-0866-14
anti-mouse CD55 PE: https://www.sinobiological.com/antibodies/mouse-cd55-daf-50468-r076-f
anti-mouse NKp46: https://www.rndsystems.com/cn/products/mouse-nkp46-ncr1-antibody_af2225
Donkey anti-goat IgG H&L-Alexa Fluor 488: https://www.abcam.cn/donkey-goat-igg-hl-alexa-fluor-488-ab150129.html
anti-mouse α-SMA: http://www.boster.com.cn/home/product/anti-sma-antibody-monoclonal-1a4_bm0002.html
Goat anti-rabbit IgG H&L-HRP: https://www.abcam.cn/products/secondary-antibodies/goat-rabbit-igg-hl-hrp-ab6721.html

Animals and other research organisms

Policy information about studies involving animals; ARRIVE guidelines recommended for reporting animal research, and Sex and Gender in Research

Laboratory animals: C57BL/6 mice (8 to 12 weeks old) were purchased from Department of Laboratory Animal Science, Peking University Health Science Center (Beijing100191, China). Il21r-/- mice were purchased from the Jackson Laboratory (stock no. 019115). In all mattings, female mice were bred with fertility-proven male mice, and the timing of conception was determined by detection of a copulation plug as gd0.5. All animals were bred and maintained in a specific-pathogen-free facility with a 12 h light/12 h dark cycle, an ambient temperature of 20–24 °C, and humidity of 30–70%.

Wild animals: This study did not involve the use of wild animals .

Reporting on sex: All the data were collected from female mice to study on the uterine NK. Male mice were used for mating. The anogenital distance and visible nipples are used to assign sex for young mice. About two hundred female mice were sacrificed for the study.

Field-collected samples: No field-collected samples were used in this study.

Ethics oversight: The animal study protocols used were approved by the Ethics Committee of Peking University Health Science Center.

Note that full information on the approval of the study protocol must also be provided in the manuscript.

Flow Cytometry

Plots

Confirm that:

- [x] The axis labels state the marker and fluorochrome used (e.g. CD4-FITC).
- [x] The axis scales are clearly visible. Include numbers along axes only for bottom left plot of group (a 'group' is an analysis of identical markers).
- [x] All plots are contour plots with outliers or pseudocolor plots.
- [x] A numerical value for number of cells or percentage (with statistics) is provided.

Methodology

Sample preparation: For uterus digestion, the tissues were harvested from virgin or pregnant mice. Then, they were opened longitudinally, cut into small pieces (~5 mm2), and incubated for 40 min at 37 °C in RPMI 1640 containing 1mg/mL collagenase type IV (Sigma-Aldrich), 0.1 mg/mL DNase I (Roche), and 5% FBS. The digested tissues were strained through a 40 μm filter, and washed with PBS containing 2% FBS, followed by antibody staining.

Instrument: Flow cytometry data were collected by LSRFortessa (BD Biosciences) and FACSAria™ III (BD Biosciences)

Software: Flow cytometry data were collected by BD FACSDiva Software and analyzed by Flowjo V10

Cell population abundance: The purities of the sorted uterine trNK were more than 95%

Gating strategy

For immune cells, first we gated the lymphocytes based on the FSC-A and SSC-A. Singlets were gated according to the pattern of FSC-H vs FSC-W and SSC-H vs SSC-W. The specific cell population was gated on the indicated markers as described in the manuscript

☒ Tick this box to confirm that a figure exemplifying the gating strategy is provided in the Supplementary Information.
